# Transcriptomic signatures in tetrapartite brain region identifies shared and unique gene signatures for substance-use

**DOI:** 10.3389/fncel.2026.1770214

**Published:** 2026-03-18

**Authors:** Avinash Veerappa, Chittibabu Guda

**Affiliations:** 1 Department of Genetics, Cell Biology and Anatomy, University of Nebraska Medical Center, Omaha, NE, United States; 2 Center for Biomedical Informatics Research and Innovation, University of Nebraska Medical Center, Omaha, NE, United States

**Keywords:** addiction, alcohol, cocaine, dependency, nicotine, opioids, reward, substance abuse

## Abstract

**Introduction:**

Chronic substance use is a neuropsychiatric disorder marked by persistent craving, reward seeking, and progression to addiction. The midbrain governs hunger, reward, and pleasure; the DLPFC modulates craving, decision making, and tolerance; the NAc influences feeding, reward, stress, and drug self-administration; and the amygdala regulates emotion and memory.

**Methods:**

To understand these complex and dynamic events in the context of substance use disorders, we profiled transcriptomes from these four regions and integrated clustering, biclustering, WGCNA, and pathway enrichment analyses.

**Results:**

Upregulation of gene expression was dominant in all four brain regions of cases versus controls. Distinct differential transcriptomic signatures were both unique to individual regions and shared across regions, identifying 186 genes exclusive to midbrain, 29 to DLPFC, 160 to NAc, and 442 in amygdala. Network analysis revealed DEGs across all regions interconnected via a neuropeptide-neurotransmitter axis, suggesting substances disrupt the equilibrium between neurotransmitters and neuropeptides. Significant upregulation of CSF3, GADD45B, SOCS3, and NPAS4 across all four regions enriched the CREB Signaling in Neurons pathway, supporting their involvement in long-lasting maladaptations of neurocircuitry due to chronic substance use.

**Discussion:**

By unraveling unique and shared transcriptomic signatures, our study advances understanding of crosstalk among key players in each brain region in substance use, implying that induction and exclusion signals drive distinct pathway signaling and sustain addiction behavior. Alongside known genes in substance biology and addiction, we also identified several novel biomarkers that could confer susceptibility for addiction risk.

## 1 Introduction

Substance use disorder (SUD) is a long-lasting neuropathological condition centered on persistent craving, pleasure, and reward, ultimately progressing to addiction (APA, 2013). The most used licit and illicit substances include opioids, nicotine, alcohol, heroin, and methamphetamine (Faller et al., 2021). Several factors are involved in the addiction process beginning from novelty/sensation seeking to dependency. The presence of personality traits for ‘novelty seeking’ and ‘impulsive sensation seeking’ is the primal factor motivating addiction (Wingo et al., 2016). These traits enable experimentation with drugs of abuse, leading to an increase in the extracellular concentration of dopamine (DA) in the brain, forming the crux of addiction. The pleasure from the experimentation stage, reward from regular use and abuse, reinforcement due to dependency, and tolerance from addiction complete the cycle of addiction. In contrast, a subgroup of users experiences a spectrum of reactions such as withdrawal, negative effects, craving, and stress sensitization while transitioning between detox, treatment, and recovery accompanied by frequent relapses. These complex and dynamic events require systematic dissection of molecular events distinct at each stage to comprehensively understand the etiology of substance addiction and the de-addiction process.

In the brain, the midbrain controls hunger, reward, and pleasure traits; the dorsolateral prefrontal cortex (DLPFC) controls craving, decision making, and tolerance traits; the nucleus accumbens (NAc) controls feeding, sexual, reward, stress-related, and drug self-administration behaviors, while the amygdala regulates stimulus to emotion, fear, and memory (Goldstein and Volkow, 2011; Lammel et al., 2014; Scofield et al., 2016). Since addiction is a consequence of the interplay among these traits, it is logical to believe the existence of interrelationship among midbrain, DLPFC, NAc and amygdala areas and the presence of spatial diffusion of information driven by transcriptomic and metabolic alterations from substance use influencing the addiction. Chronic substance use has the propensity to build maladaptive circuits inspired via the constant activation of pro-reward circuits during the development and progression of addiction.

In a previous study, we established a ‘core addictome’ pathway based on the genes identified from genetic, epigenetic, and transcriptome studies (Veerappa et al., 2021). In the current study, we used RNA sequencing data from four human brain regions that include midbrain, DLPFC, NAc, and amygdala tissues from opioid and polysubstance users to achieve the following goals: (a) assess concordant and discordant gene expression among the midbrain (*n* = 50), DLPFC (*n* = 153), NAc (*n* = 80) and amygdala (*n* = 42) regions of polysubstance users, (b) map expression signatures and identify cross talks resulting from spatial diffusion of information, and (c) identify potential region-specific therapeutic targets for substance use and addiction. By unraveling the expression differences and similarities between and across these groups, this study advances the understanding of substance use biology and provides potential new genes and markers specific to four distinct regions of the human brain.

## 2 Methods

### 2.1 Data access and pre-processing

Gene expression data in paired-end FastQ format from the following four studies were downloaded without restrictions from the Sequence Read Archive (SRA). Midbrain cohort with *n* = 50 (cases = 30 and controls = 20) bearing accession identifier (ID) PRJNA492904 was obtained from a study involving differential gene expression networks in opioid users (Saad et al., 2019), while DLPFC-chronic and NAc cohort data with *n* = 80 (cases = 40 and controls = 40) bearing accession ID PRJNA729761 was obtained from a study aiming at understanding transcriptional dynamics of neuroinflammation and synaptic remodeling in opioid users (Seney et al., 2021) (Table 1) (Supplementary Figure 1). Data on the amygdala cohort with *n* = 42 (cases = 21 and controls = 21) bearing accession ID PRJNA800378 studying the transcriptional alterations in amygdala regions in opioid users (Carmack et al., 2022) (Table 1) (Supplementary Figure 1). We downloaded the metadata, and raw FastQ files containing RNA-seq reads using the *nf-core/fetchngs pipeline v.1.3* on *Nextflow* v21.04.0 (Ewels et al., 2020; Patel et al., 2021a). The sample ID list to be extracted from the study was provided in the command, which was resolved back to appropriate experiment-level ids to be compatible with the ENA API. Available metadata for all sample IDs, including download links to FastQ files, were extracted via ENA API. FastQ files were downloaded in parallel via *curl*, and a *md5sum* check was performed to verify their integrity. Metadata of all samples and paths to their respective FastQ files were collated in a single sample sheet for performing RNA-seq quantification and calling variants (SNVs and Indels) using *GATK* best practices workflow for RNAseq short variant discovery. All cases were primarily diagnosed with OUD/opioid dependence, although polysubstance use was common across cohorts. Because co-use of other substances was frequent and not consistently quantifiable across datasets, we could not control for polysubstance exposure in downstream analyses; therefore, we grouped these cases under the umbrella term “substance use disorder”.

**Table 1:** Summary of tissue origin, demographics and specimen quality from opioid abuse subjects.

	Midbrain	Midbrain controls		DLPFC and NAc	DLPFC and NAc controls		Amygdala	Amygdala controls
Sample size	*n* = 30	*n* = 20	Sample size	*n* = 40	*n* = 40	Sample size	*n* = 21	*n* = 21
Age	51.5 ± 1.03	50.4 ± 0.93	Age	46.9 ± 7.3	47.3 ± 9.5	Age	46.3	50.2
Race/sex			Sex			Sex		
Black male	22 (73%)	14 (70%)	Male	10	10	Male	*n* = 11	*n* = 16
White male	8 (27%)	6 (30%)	Female	10	10	Female	*n* = 10	*n* = 5
Brain pH	6.53 ± 0.03	6.60 ± 0.03	Race			Race		
RIN	7.25 ± 0.09	7.39 ± 0.11	Black	1	7	Blacks	1	5
			White	19	13	Whites	18	16
			Brain pH	6.4 ± 0.2	6.6 ± 0.3	Asian		
			RIN	7.8 ± 0.7	8.0 ± 0.7	Autolysis	17.5	19.0

RIN, RNA integrity number; PMI, Postmortem Interval.

### 2.2 RNASeq quantification

We quantified gene expression on the *Nextflow* v.21.04.0 framework (Ewels et al., 2020) using the *nf-core/rnaseq* pipeline (Patel et al., 2021b). Before quantification, we performed a series of Quality Control (QC) procedures using *FastQC 0.11.9* (Simon, 2021) to detect adapter sequences, contaminants, and overrepresented sequences but found none. The *extract* command of *UMI-Tools* removed random nucleotides that were attached to the start of reads. Adapter trimming was performed using the 13 bp (AGATCGGAAGAGC) Illumina standard sequencing adapters by *Trim Galore! v.0.6.6.* (Krueger, 2021) while ribosomal RNA was removed using *SortMeRNA v.4.3.4* (Kopylova et al., 2012). The cleaned-up FastQ reads were used for alignment against the human reference genome, GRCh38, using the *STAR v.2.6.1d* (Dobin et al., 2013). The aligned reads were sorted and indexed using *SAMtools v.1.12* (Li, 2011). The *dedup* command from *UMI-Tools* was used to remove PCR duplicates, and for every group of duplicate reads, a single representative read was retained. Further filtering to locate and tag duplicate reads arising during library construction was performed using the *MarkDuplicates* tool from *Picard v.2.23.9*. *StringTie v.2.1.7* (Pertea et al., 2015) was used for assembly and quantitating full-length transcripts representing multiple splice variants for each gene locus. Extensive post-alignment QC was performed using *RSeQC v.3.0.1*, *Qualimap v.2.2.2-dev*, *dupRadar v.1.18.0*, *Preseq v.3.1.1*, and *DESeq2 v.1.28.0* (Love et al., 2014) to review sequence quality, nucleotide composition bias, PCR bias, and GC bias, and to evaluate sequencing saturation, mapped reads distribution, coverage uniformity, strand specificity, and transcript level RNA integrity. We quantified the transcript and expression with *Salmon v.1.4.0.* (Patro et al., 2017) using the GENCODE v.38 reference transcript annotations. Lastly, raw reads, alignments, gene biotype, sample similarity, and strand-specificity checks were done using *MultiQC* (Ewels et al., 2016) (Supplementary Figures 2, 3).

### 2.3 Differential gene expression (DGE)

DGE analysis was performed based on statistical modeling of counts using *DESeq2* utilizing gene-level read count data. Low expressed genes with ≤0.5 counts per million (CPM) were filtered. CPMs were calculated by normalizing the read counts by the total counts per sample using *edgeR* (Robinson et al., 2010). Transformation of counts data for clustering analysis and principal component analysis (PCA) was carried out using *edgeR*. *ANOVA* detected bias in sequencing depths (as *P* was found <0.01) among the samples as a potential confounding factor, but the ratio of the maximum to minimum total counts was within 1.5 among groups; therefore, it was accepted and retained for further analysis. Hierarchical clustering was performed on the transformed data and visualized with a heatmap. Genes across all samples were ranked by the standard deviation (SD), and the topmost 500 genes were used in the *heatmap.2* function. The same group of genes were then used for unsupervised k-means clustering based on their expression pattern across all samples. The number of clusters was adjusted to 4, and enrichment analysis was conducted on each cluster. The student’s *t*-test was used to compare the scores across the clusters, and the *p*-values were corrected for multiple testing using false discovery rate (FDR). Linear transformation of the data was done via principal component analysis (PCA) to assess the direction of the most variation among samples. PCA enables us to project samples into two-dimensional space. We also treated the PCA onto each gene as expression data to run pathway analysis with the PGSEA package. This runs the PAGE algorithm for each pathway, which performs a one-sample *t*-test on each gene set in the ‘biological processes’ category of Gene Ontology (GO). The adjusted *p*-values were used to rank the pathways for each of the first five principal components.

The pathways were labeled first with FDR, followed by the principal components. Multidimensional scaling (MDS) was used to project data points in high-dimensional spaces into 2D surfaces while preserving the distances as much as possible. Pathway analyses were performed based on fold-change using *GSEA* (Gene Set Enrichment Analysis). Biclustering was executed to detect clusters of genes correlated among a subset of samples. We used the default settings in the *biclust R* package (Kaiser, 2011) and utilized the different algorithms for biclustering such as *QUBIC* and runibic in addition to *BCCC*, *BCXmotifs*, *BCPlaid*, *BCSpectral*, *BCBimax*, and *BCQuest*. To visualize correlation in gene expression, we used *WGCNA* (weighted correlation network analysis) to identify co-expression networks from normalized gene expression data. GO (Gene Ontology) analyses were performed on the modules from co-expression networks.

### 2.4 Pathway analysis

Pathway analysis was performed using *GSEA* (Gene Set Enrichment Analysis) (Subramanian et al., 2005), *PAGE* (Parametric Analysis of Gene Set Enrichment) (Kim and Volsky, 2005), *PGSEA* package (Furge and Dykema, 2012) and *ReactomePA* (Reactome Pathway Analysis), (Yu and He, 2016). Besides these, Ingenuity Pathway Analysis (IPA) was also used to generate networks and pathways (a more detailed explanation follows).

### 2.5 Integrated pathway analysis (IPA)

Pathway analysis was performed using IPA (Kramer et al., 2014) on differentially expressed genes (DEGs) to identify enriched biological processes, canonical pathways, upstream transcriptional regulators, and gene networks. The predicted state, Z-score, and *p*-value enabled us to identify regulators of interest. Downstream Effects Analysis, Causal Network Analysis, and Molecule Activity Predictor (MAP) were performed on the datasets to ascertain the biological significance of genes and proteins. Pathways were constructed by utilizing the *grow and connect* function. Experimental evidence-backed and tissue-specific activations, inhibitions, protein–protein interactions, protein-RNA interactions, and protein-DNA interactions were overlayed on the resulting network to understand the crosstalk between DGEs and variant-carrying genes. Further, IPA was also used to perform DGE comparison analysis, upstream regulatory analysis, and mechanistic network analysis.

## 3 Results

In this study, we utilized RNA sequencing data from postmortem brain samples from opioid-intoxicated individuals and matched controls, focusing on four critical regions: the midbrain, DLPFC, NAc, and amygdala. The sequencing data in FastQ files was sourced from a demographically well-balanced cohort (median age 46.5, with 68% male and 32% female participants) across all regions, as detailed in Table 1.

### 3.1 Differential gene expression analysis in midbrain, DLPFC, NAc, and amygdala brain regions

**Figure 1:**
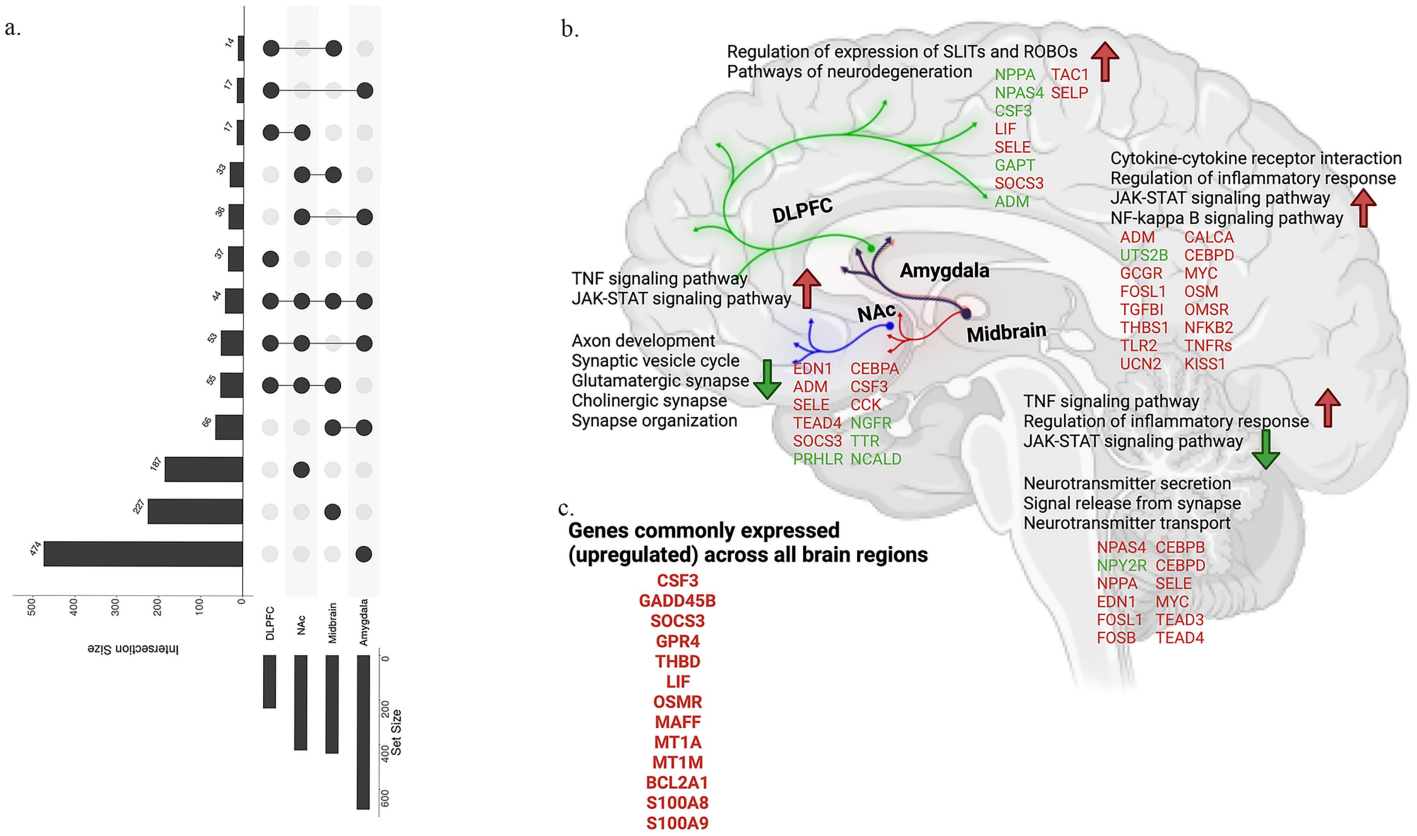
Shared genes and pathways between tetrapartite regions. **(a)** Upset plot representing shared and unique genes between four distinct regions of midbrain, DLPFC, NAc, and amygdala. **(b)** Shows the dysregulated pathways and genes in the tetrapartite regions. Genes in red are upregulated, and green are downregulated.

In our comprehensive transcriptomic analysis across four key brain regions—the midbrain, DLPFC, NAc, and amygdala—we observed notable patterns of gene expression relevant to SUDs. Utilizing a library of 57,773 transcripts, our filtered datasets revealed 22,671, 26,118, 27,587, and 25,525 transcripts in the midbrain, DLPFC, NAc, and amygdala cohorts, respectively. Differential gene expression (DGE) in each brain region between control and cases was assessed using DESeq2, after incorporating covariate regression for age, sex, brain pH, and RNA integrity number (RIN). Using log2 fold change cutoff of ≤ −1 or ≥ 1, we identified a total of 329 DEGs from the midbrain, of which 310 genes were upregulated, and 19 downregulated. Some of the notable upregulated DEGs were EDN1, NPPA, NPAS4, MYC, FOSL1, FOSB, NR4A3, CSF3, OXTR, TEAD3/4, while NPY2R was found downregulated. On the other hand, DLPFC region showed 123 DEGs, among which 106 were found to be upregulated, and 17 downregulated. Genes belonging to the former group include NPAS4, EGR2, and TDGF1, while some of the genes belonging to the latter group include CSF3, SOCS3, CEBPD, ADM, LIF, HAMP, and SELE. Comparatively, similar analysis in the NAc cohort identified 292 genes, of which 171 were upregulated and 121 were downregulated. Some notable DEGs in this region include NPAS1, LIF, EDN1, HAMP, CEBPA, CSF3, CHRNA6, SOCS3, NGFR, ADM, NCALD, SSTR3, SELE, SELP, and NXPH4. Lastly, amygdala region showed a higher number of DEGs (635 genes), of which 181 were downregulated and 454 upregulated. In this region, MYC, SOCS3, ADM, C1R, CSF3, FOSL1, GADD45A, and GADD45B were found to be upregulated. Annotations of all DEGs are provided in Supplementary Table 1. Assessing the uniqueness and commonalities among the DEGs across these four regions, identified 227 genes exclusive to the midbrain, 37 genes exclusive to DLPFC, 187 genes to NAc, and 474 genes for amygdala regions (Figure 1a). However, 44 genes were identified to be commonly differentially expressed across all the four regions (Supplementary Table 2). The list of DEGs from all regions is provided in Supplementary Table 3. Pathways found differentially up- and down-regulated across the four regions of the brain are compared in Figure 1b.

### 3.2 Integrative biclustering and K-means analyses reveal key neuronal pathways in SUDs across brain regions

**Figure 2:**
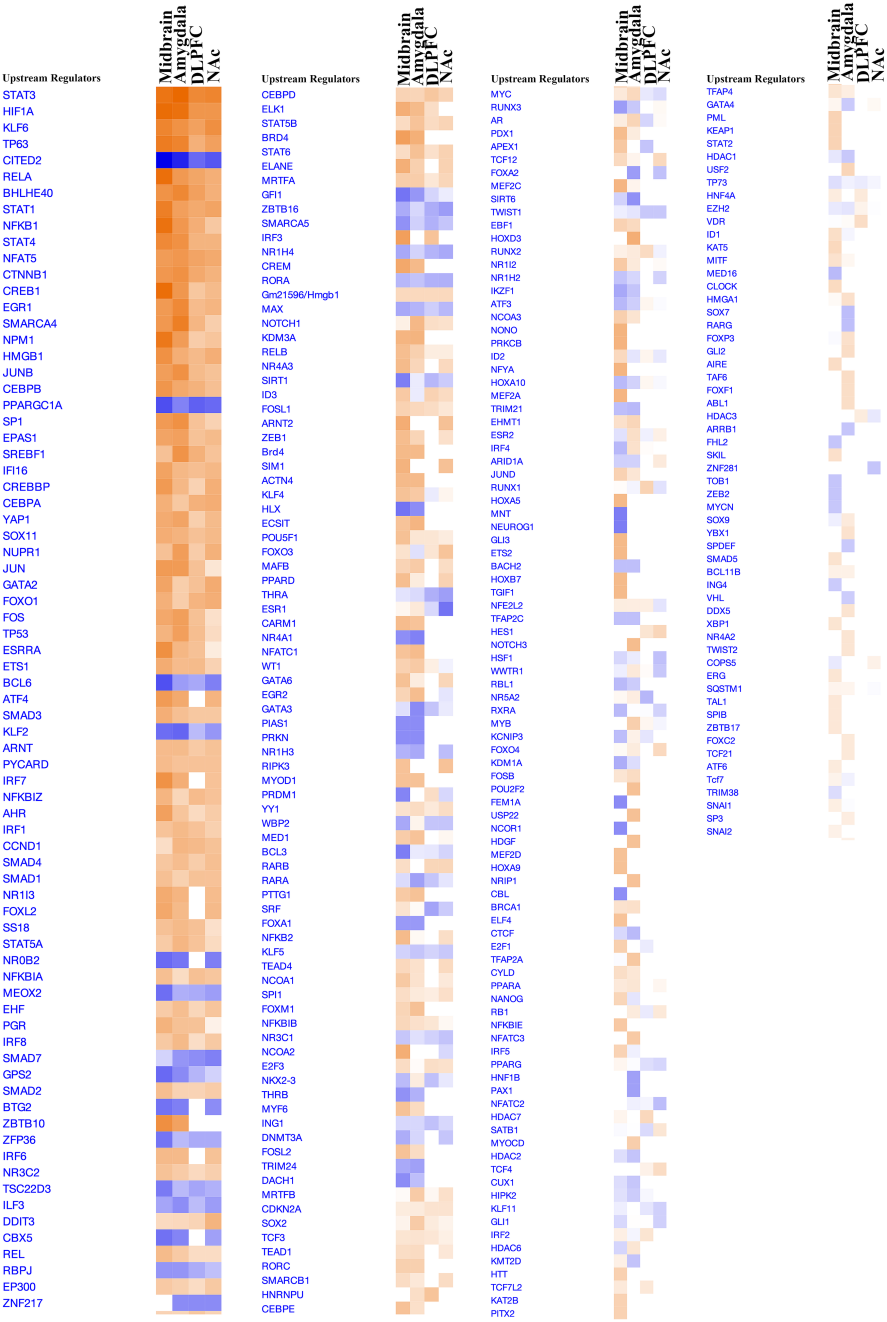
Enrichment analysis performed on biclusters using IPA, GO, KEGG, WikiPathways, ReactomePA, and TF databases on midbrain, DLPFC, NAc, and amygdala.

Employing biclustering on the top 1,000 most variably expressed genes, we uncovered gene correlations specific to subsets of opioid cases and controls in each brain region. This analysis incorporated biclustering methodologies such as BCCC, QUBIC, runibic, BCXmotifs, and BCPlaid, alongside cross-referencing with a comprehensive suite of databases including IPA, GO, KEGG, WikiPathways, ReactomePA, and others (Figure 2). Figure 2 summarizes the signaling pathways identified across all four brain regions. We observed widespread gene upregulation in cases vs. controls and both shared and region-specific signatures (midbrain 186 unique genes, DLPFC 29, NAc 160, amygdala 442). Network mapping showed DEGs interlinked by a neuropeptide-neurotransmitter axis, consistent with disrupted transmitter balance in chronic substance use. Across regions, we observed a strong neuroimmune signal, with cytokine-cytokine receptor interaction enriched in all four regions and additional TNF, NF-κB, JAK–STAT, and IL-17 modules recurring in multiple regions. Synaptic and axonal remodeling pathways were enriched but varied by region; NAc uniquely favored innate-immune/NETs modules, while the amygdala uniquely engaged p53/MAPK/ECM-microRNA programs. Notably, CSF3, GADD45B, SOCS3, and NPAS4 were upregulated in all regions, enriching CREB signaling in neurons and implicating long-lasting neurocircuit adaptations and potential biomarkers of addiction risk (Figure 2). In parallel, k-means clustering on variably expressed genes, constrained to four clusters, and normalized by mean centering, delineated distinct gene clusters for each brain region (Supplementary Table 4). Utilizing the KEGG pathway database, we identified pathway enrichments for each cluster, with a focus on SUDs-related traits. Notable enrichments included Cocaine and Nicotine addiction pathways, along with several neurotransmitter synapse pathways like GABAergic synapse (1.2e−03), Serotonergic synapse (9.4e−05), Cholinergic synapse (7.9e−03), and Synaptic vesicle cycle (1.9e−04), reflecting a broad engagement of these pathways in SUDs across all studied brain regions (Supplementary Table 4).

### 3.3 Elucidating transcriptional regulators and predictive upstream influences in brain regions

**Figure 3:**
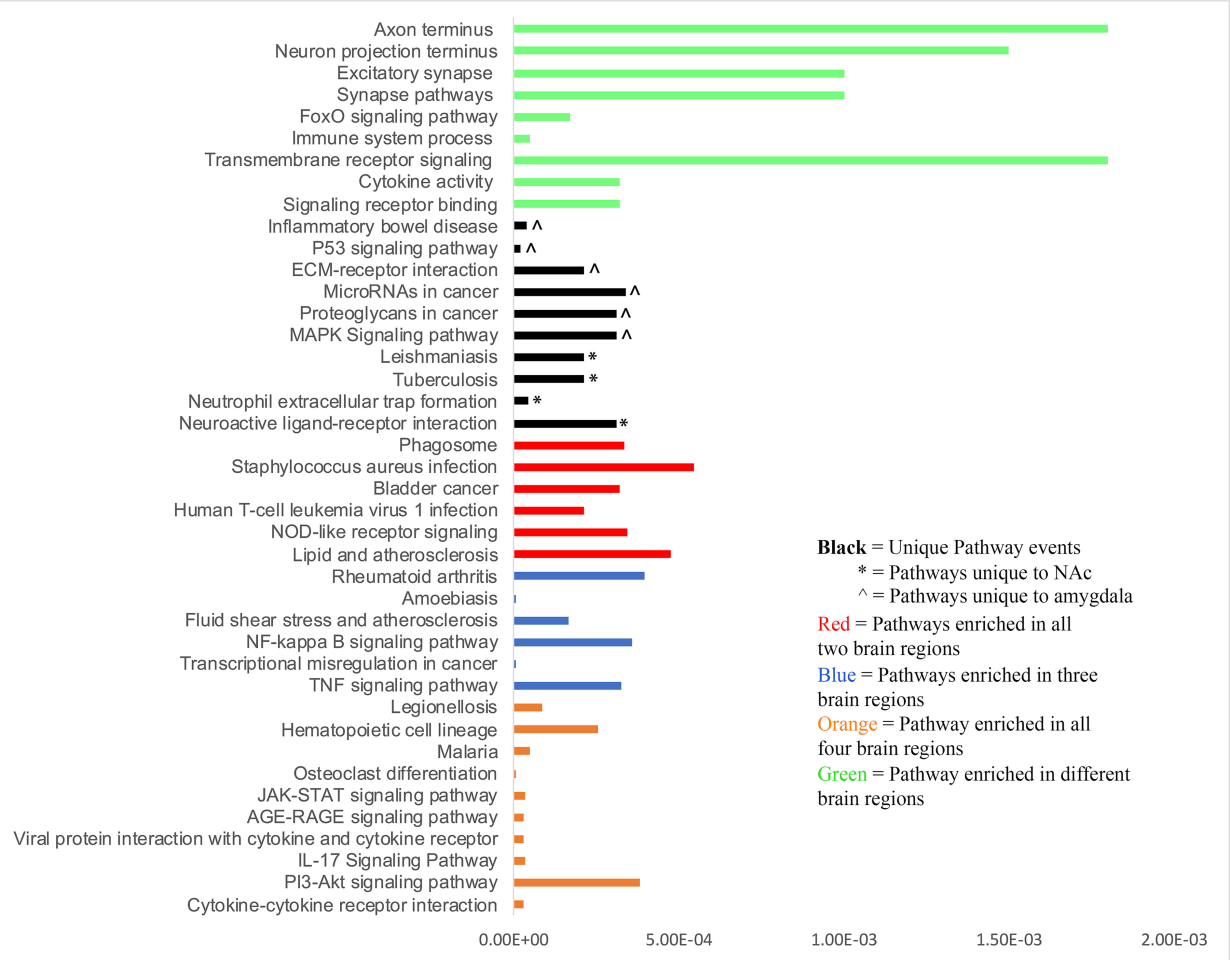
Upstream regulator analysis performed on DEGs using IPA identified a panel of potential regulators. The upset plot displays the number of concordant and discordant TF activations and inhibitions across four brain regions.

Utilizing Upstream Regulator Analysis (URA) via IPA, we identified key transcriptional regulators in the midbrain, DLPFC, NAc, and amygdala, based on DEGs and a z-score threshold of ±2 (Figure 3). The analysis revealed distinct sets of transcription regulator genes: 31 in the midbrain, 8 in the DLPFC, 14 in the NAc, and 32 in the amygdala. Moreover, URA predicted an array of potential upstream regulators not classified as DEGs, amounting to 178, 409, 354, and 468 in the respective regions, as detailed in Supplementary Table 5. Significantly, this study identified a panel of regulators including JUN, JUNB, FOS, and FOSL1, which are early-expressing genes in SUDs. Notably, 69 and 27 TFs had positive and negative activation Z socres, respectively, across the four brain regions. The z-scores for these regulators ranged from −4.370 to 4.829, highlighting the variability of potential regulatory influences (Supplementary Table 6). The summary table (Supplementary Table 7) lists the top TFs and other regulators alongside their corresponding DEG targets across all four brain regions. In addition, Supplementary Figure 4 illustrates the predicted mechanistic interactions between these regulators and the affected DEGs.

### 3.4 Differential gene enrichment and pathway regulation in brain regions revealed by WGCNA

**Figure 4:**
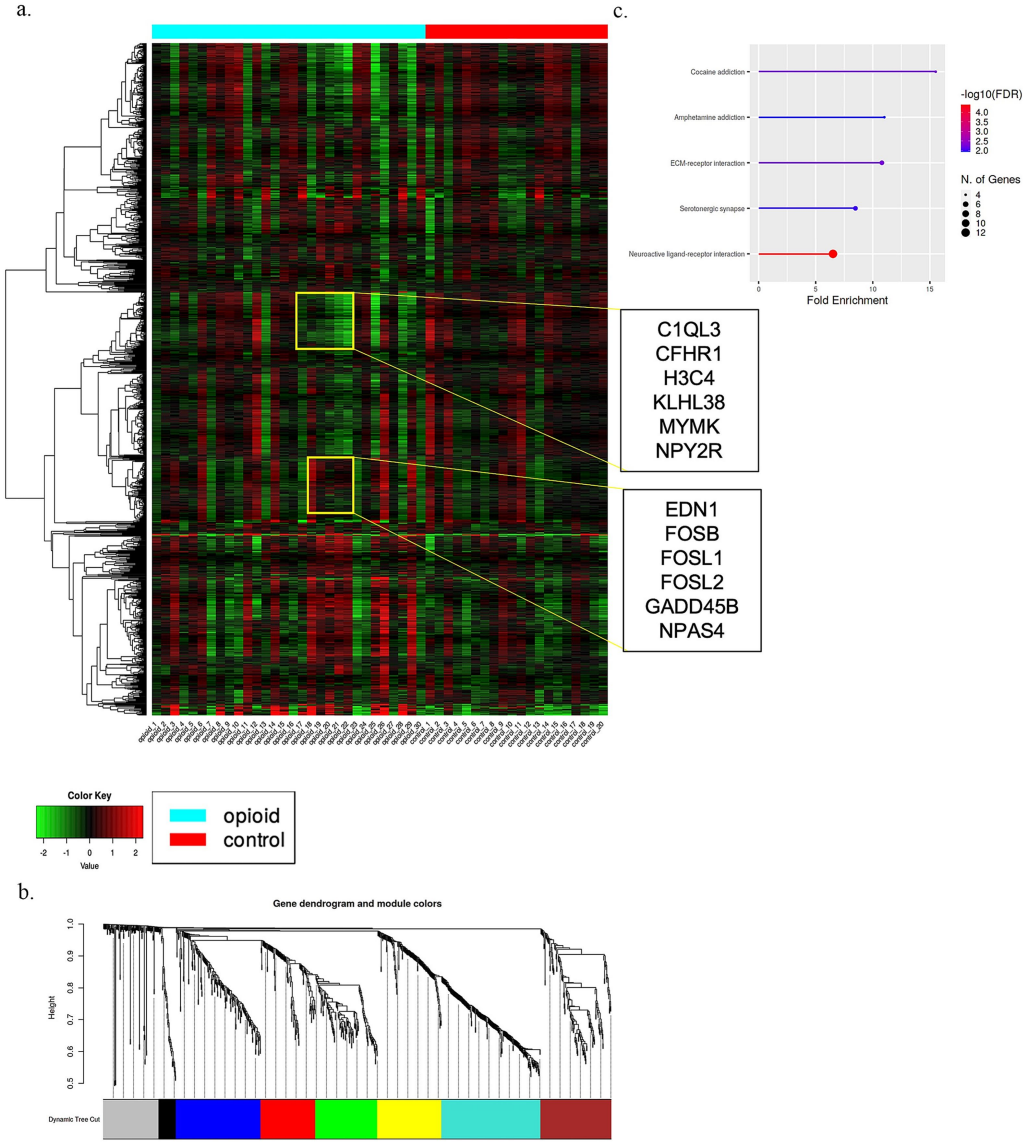
Shows gene expression signatures of midbrain cohort. **(a)** Is heatmap, **(b)** is the gene dendrogram and respective module colors, and **(c)** is the lollipop plot generated based on enriched pathways on WGCNA modules.

Employing WGCNA, we constructed co-expression networks and sub-modules for each brain region, using the most variably expressed genes from the opioid cases and controls. The resulting networks, segmented into color-coded modules, revealed distinct patterns of gene enrichment. In the midbrain (Figure 4a), neuropeptide activation (adj. *p*-value 1.6e−05), serotonergic synapse (2.3e−07), and addiction pathways (amphetamine/cocaine: 8.7e−04) were prominent (Figures 4b,c; Supplementary Table 8). The DLPFC cohort exhibited networks primarily related to immune functions and synapse pruning (Supplementary Figure 5, Supplementary Table 9). The NAc region displayed significant enrichments in neuropeptide hormone activity, addiction pathways, neurotransmitter transport, and various synaptic functions (Supplementary Figure 6, Supplementary Table 10). In contrast, the amygdala cohort was enriched for neuropeptide activations, TNF signaling, and Toll-like receptor pathways (Supplementary Table 11). A gene dendrogram and its corresponding lollipop plot depicting the pathways in nine modules along with network for the amygdala region is shown in Supplementary Figure 7.

**Figure 5:**
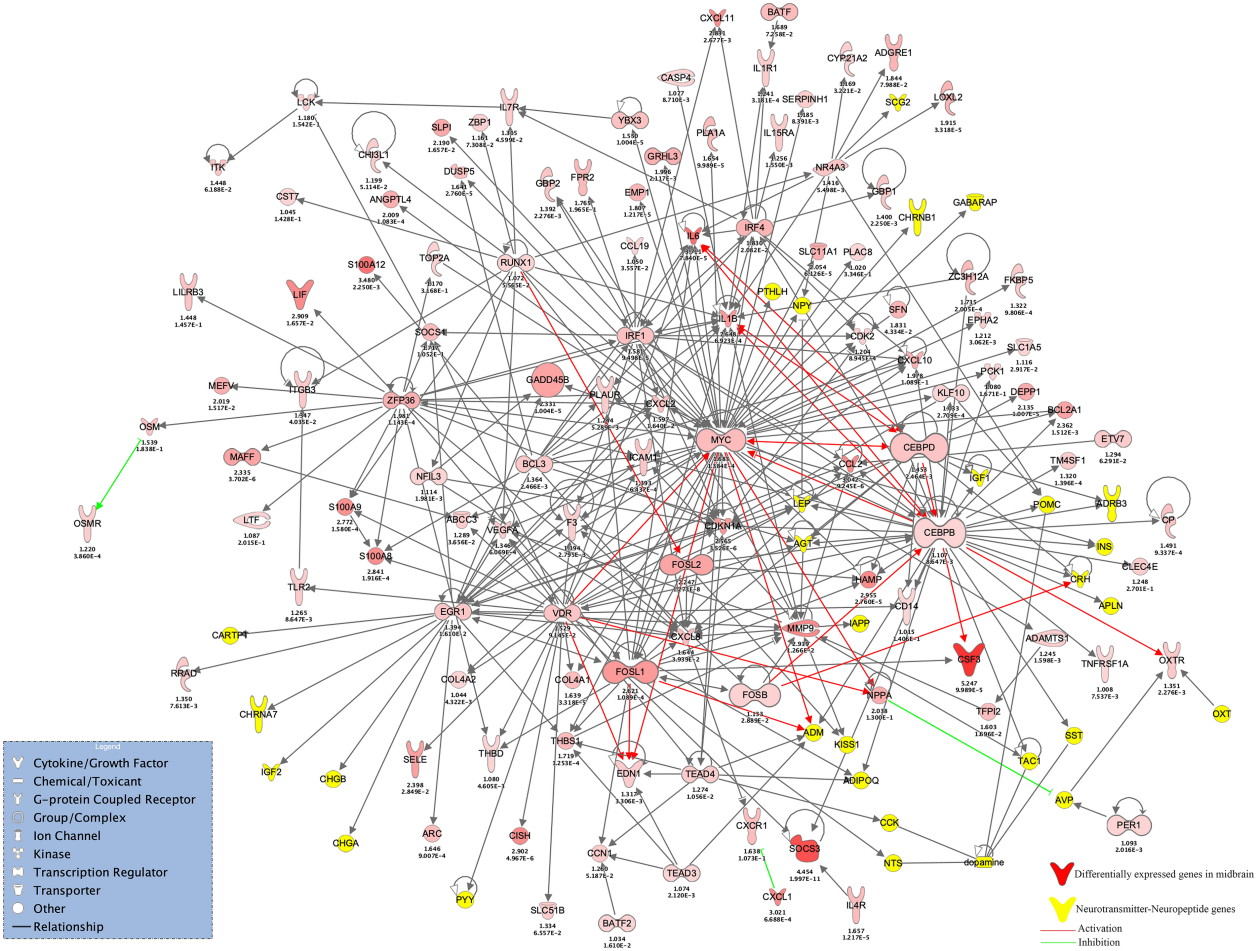
Canonical pathway enrichment for all four brain regions based on *z*-score activation.

Further pathway enrichment analysis across these regions, using methods like GAGE, GSEA, PGSEA, and ReactomePA, with an adj.pval cut-off of 0.05 identified unique patterns of pathway regulation (Supplementary Tables 12–15). Downregulation in nerve impulse propagation and neurotransmitter release pathways contrasted with upregulation in inflammatory and immune response pathways in the midbrain. The DLPFC and amygdala regions showed upregulation in canonical signaling pathways, while the NAc region exhibited downregulation in synapse organization and axonogenesis. Across all regions, IPA analysis highlighted consistently activated pathways including wound healing, IL-17 signaling, and CREB Signaling in Neurons (Figure 5, Supplementary Table 16).

### 3.5 Regulatory interactome of DEGs from tetrapartite regions

**Figure 6:**
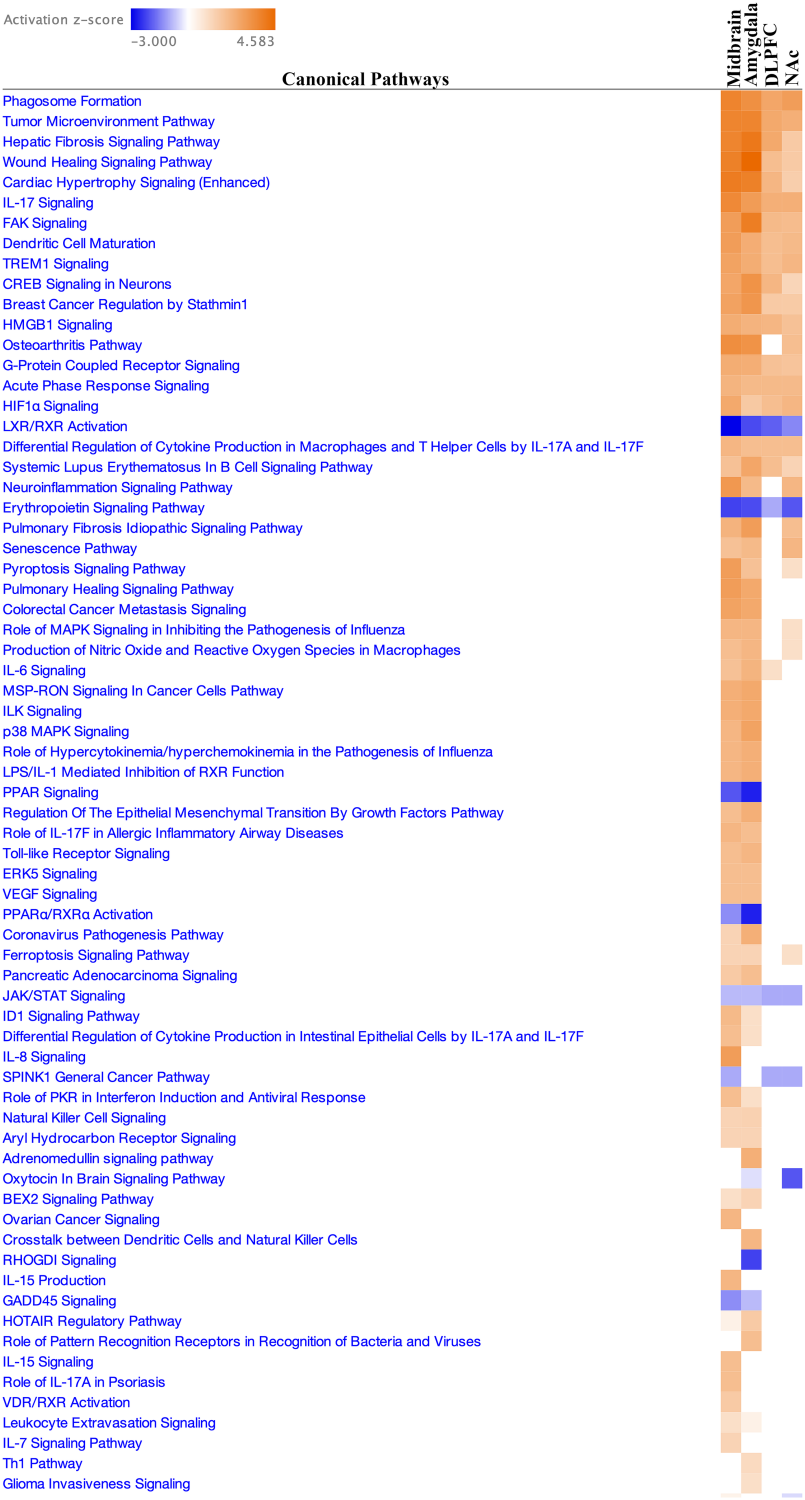
Shows regulatory network of the midbrain region established using IPA. This network shows DEGs flanked by a spectrum of genes belonging to a family of neuropeptides, neurotransmitters, and their targets. The midbrain network was centered on several differentially upregulated transcription regulators, such as MYC, FOSB, FOSL1, FOSL2, VDR, TEAD4, EGR1, NFIL3, RUNX1, ZFP36, YBX3, KLF10, IRF1, IRF4, and BCL3. The midbrain DEGs also contained neuropeptides EDN1 and NPPA that were found upregulated. Neurotransmitters and their targets emerged to cluster around the network, with few molecules from each target appearing to behave as gateway molecules to their respective neurotransmitter clusters. MYC was found to bind and regulate neuropeptides ADM, CCK, LEP, NPY, PTHLH, GRP, NAMPT, and INS. FOSB was found to regulate CRH, while FOSL1 was found to regulate ADM, NTS, and APLN, whereas FOSL2 was found to regulate LEP and KISS1. VDR, on the other hand, regulated LEP, AGT, ADIPOQ, PYY, GABARAPL1, and GABARAPL2. In the DEGs network, several transcription regulators were found significantly upregulated and located central to the network, such as CEBPB, CEBPD, EGR1, FOSB, FOSL1, FOSL2, BATF, ZFP36, IRF1, IRF4, VDR, BCL3, NFIL3, RUNX1, and YBX3.

Utilizing IPA, we constructed regulatory interactomes of DEGs across four brain regions to decipher unique and shared neuronal maladaptations. In the midbrain, DEGs revealed a broad spectrum of neuropeptides, neurotransmitters, and their targets, including notably upregulated EDN1 and NPPA (log2-fold increases of 1.31 and 2.03, respectively). Central to the DEGs network, key transcription regulators like CEBPB, EGR1, FOSB, and MYC were significantly upregulated, orchestrating a complex regulatory network (Figure 6). These regulators play a pivotal role in modulating the neuropeptide and neurotransmitter axis, with MYC regulating a range of neuropeptides such as ADM and NPY, and FOSB targeting CRH (Figure 6). Additionally, VDR and TEAD4 were identified as crucial for regulating LEP and CCK, among others. Notably, the network identified gateway molecules facilitating cluster interactions, emphasizing the intricate regulatory relationships. Downregulated genes, such as NPY2R and NPAS4, highlighted disruptions in neuropeptide signaling, with NPAS4’s downregulation affecting BDNF and neurotransmitter systems like glutamate and serotonin. This analysis sheds light on the intricate regulatory mechanisms underlying brain region-specific maladaptations, with significant implications for understanding the molecular basis of neurological disorders.

In the DLPFC, a network of DEGs intricately interacts with neuropeptides and DA targets, highlighting a complex interplay pivotal in SUDs. Notably, the neuropeptides EDN1 (log2fc 1.37) and ADM (log2fc 2.35) were identified as differentially expressed, initiating diverse interactions with other neuropeptides and neurotransmitters including EDN3, NPPB, and serotonin, thereby influencing a broad network of molecules involved in SUDs (Supplementary Figure 8). Moreover, CCL2’s interaction with serotonin underscores its potential role in these networks. The regulatory landscape further revealed EGR2, a transcription regulator downregulated, as a key node controlling numerous neuropeptides and neurotransmitters, affecting targets such as CHRNA7 and GRIN2A, which in turn modulate several DEGs (Supplementary Figure 8). Central to the DEG network, CEBPD and ZFP36 emerged as significant hubs. CEBPD regulates a suite of neuropeptides including CRH and neurotransmitter receptors like ADRB3, alongside other DEGs (BCL2A1, CD14), and is bidirectionally influenced by IL6 and IL1B (Supplementary Figure 8). Conversely, ZFP36 focuses on regulating DEGs like LIF and SPP1, which subsequently impact neuropeptides and DA targets, including DRD2 (Supplementary Figure 8).

In the NAc, a distinctive profile emerged with only four neuropeptides and one neuropeptide target exhibiting differential expression among the genes studied. Specifically, ADCYAP1 and NXPH4 were downregulated (log2fc − 1.26), while ADM and EDN1 showed upregulation (log2fc of 1.62 and 1.41, respectively). The target receptor PRLHR experienced a downregulation of −1.5 log2fc, highlighting a nuanced regulatory landscape (Supplementary Figure 9). Central to the NAc’s regulatory network, TFs TEAD4, CEBPA, and ZFP36 orchestrated the gene expression. TEAD4 emerged as a pivotal regulator, modulating neuropeptides such as EDN1 and ADM alongside CEBPA, which itself was downregulated by −1.03 log2fc. CEBPA influenced a broad array of targets, from neuropeptides like AGT and LEP to transcription regulators NFIL3 and OTX2 (Supplementary Figure 9). ZFP36, without directly targeting neuropeptides, impacted the network through the regulation of receptor clusters and was linked to a range of differentially expressed genes including MEFV, showcasing its regulatory breadth. The network further integrated genes like SOCS3 and CSF3, tying them to neuropeptide regulation, indicative of the complex interplay across the neurotransmitter spectrum. Notably, cholinergic and adrenergic receptor clusters were modulated by specific DEGs, illustrating the network’s influence on neurotransmitter systems. The dopaminergic receptor cluster, under the sway of POMC regulated by OTX2, underscores the intricate regulatory dynamics (Supplementary Figure 9). Mir-637, central yet not depicted in the network, regulated a suite of genes including LIF and HAMP, alongside NPAS1, a neuronal PAS domain protein notable for its consistent differential expression in opioid users across brain regions. Although NPAS4’s differential expression in the NAc did not reach the log2fc threshold of 1, its involvement highlights a broader neuronal regulatory context.

In the amygdala, the network density mirrored that observed in the other three brain regions, characterized by a similar number of DEGs around the axis of neuropeptides and neurotransmitters, as illustrated in Supplementary Figure 10. The transcription factor MYC, upregulated by 1.42 log2fc, emerged as a central regulator, influencing the expression and functionality of key molecules including EDN1, FOSL1, CEBPD, THBS1, and GRHL3. Notably, MYC itself was subject to upregulation by a cohort of regulators such as CD44 (1.35 log2fc), IL6 (3.28 log2fc), NFKB2 (1.17 log2fc), underscoring a complex regulatory feedback loop (Supplementary Figure 10). Further extending the regulatory network, TEAD4 (1.55 log2fc), SNAI1 (1.35 log2fc), and YBX3 (1.65 log2fc) propagated the signaling cascade initiated by MYC. Additionally, the ADM neuropeptide, experiencing a substantial upregulation of 2.12 log2fc, was under the regulatory influence of FOSL1, TEAD4, and MYC, highlighting a pivotal role in the amygdala’s regulatory framework. Moreover, CSF3, with a notable upregulation of 2.40 log2fc, was identified as being regulated by FOSL1, and in turn, affected downstream neuropeptide effectors such as EDN1 (Supplementary Figure 10). This intricate interplay of transcription factors and neuropeptides delineates a complex regulatory network in the amygdala, emphasizing the sophisticated molecular mechanisms underlying its function.

## 4 Discussion

### 4.1 Shared genes across midbrain, DLPFC, NAc, and amygdala limbic regions

Comparative analysis of gene expression among the four regions of midbrain, DLPFC, NAc and amygdala, identified 44 genes that commonly expressed across all four regions (Supplementary Figure 11, Supplementary Table 17). Intriguingly, all these 44 genes were found upregulated in all four regions. Some of the notable genes in this list were CSF3, SOCS3, LIF, CCL2, GPR4, THBD, THBS1, MT1A, MT1M, BCL2A1 and GADD45B, among other lesser-known candidates like ANGPTL4, CISH, DEPP1, EMP1, FPR1, MAFF, IL6, IL1B, and HAMP (Supplementary Figure 11). Some of these genes were found to be part of the Jak–STAT signaling pathway (adjP = 7.12e−08). However, CD14, CP, EDN1, among others were found to be common across only the midbrain, DLPFC and NAc regions. CCL2 protein has been shown to increase the expression of mouse Cebpb protein (Younce et al., 2009). Wakida et al. (2014) demonstrated that activation of DA neurons via the CCL2-CCR2 axis played a crucial role in enhancing reward-system activity while establishing psychic dependence on methamphetamine. With regards to CD14, CEBPB protein was found to increase the expression as well as activation of CD14 (Matsumoto et al., 1998; Pan et al., 1999). CD14 is an innate immune signaling protein that has also been regarded as an important transducer for regulating drinking behavior (Crews, 2012).

Studies have reported upregulation of colony stimulating factor (CSF) in both DLPFC and NAc regions of the brain. However, we report here the upregulation of CSF for the first time from the midbrain and amygdala regions. Calipari et al. (2018) identified the role of CSF in promoting neural responses to substances like cocaine, and their study also regarded CSF as an effector for both neural and behavioral responses. Impulsivity trait has been associated as a risk factor for susceptibility towards substance use (Kozak et al., 2019), while elevated CP (ceruloplasmin) levels have been associated with higher impulsivity in people with neuropsychiatric disorders (Bakeberg et al., 2020). In the current study, we identified upregulation of CP in midbrain, DLPFC and NAc regions, probably indicating the participation of the brain regions in imparting continuous activation state of impulsiveness in chronic substance users. GADD45B is one of the well-studied genes with evidence from the transcriptomic, epigenetic, knockdown, and knockout studies that have provided several layers of evidence towards its participation in the intersection of DA neurotransmission and substance-induced plasticity (Di Chiara and Imperato, 1988; Day et al., 2007; Ma et al., 2009; Puckett and Lubin, 2011; Sultan and Sweatt, 2013; Walker and Nestler, 2018; Campbell and Wood, 2019; Zipperly et al., 2021). Most studies have looked at the role of GADD45B in the context of NAc; however, the current study identifies significant differential upregulation of GADD45B across all the four brain regions signifying that its effect is more widespread than previously thought and is not exclusive to the NAc region. Similarly, the finding of upregulation of GADD45B in all four regions opens a new paradigm on DA presence and its role in inducing the expression of GADD45B mediated by DRD receptors.

### 4.2 MAFF, YBX3, BCL2A1, and ZFP36 upregulate the 44 genes across tetrapartite brain

Furthermore, we identified transcription regulators, MAFF, YBX3, BCL2A1, and ZFP36 expressing in all four regions, consequently, indicating the regulation of their downstream targets. Interestingly, most of these 44 genes were found to have extreme regulatory and protein–protein interactions connectivity among themselves indicating that they are actively involved in cross-talks (Supplementary Figure 11a). Besides having cross-talks among themselves, these genes were also flanked by neuropeptides and neurotransmitters and were found to exhibit activations, inhibitions, expression, protein–protein binding, and feedback loops with them (Supplementary Figure 11b). It was important to note that the following genes which are part of the 44 commonly expressing genes play crucial roles in modulating SUDs traits. CSF3 plays a role in altering the motivation (Bart et al., 2021); GADD45B in reward memory (Zipperly et al., 2021); SOCS3A in potentially establishing tolerance (Reese et al., 2019); and EDN1 in vasoconstriction (Nishiyama et al., 2017). Significant upregulation of CSF3, GADD45B, and SOCS3, was found in all four brain regions studied, while EDN1 was found upregulated in only the midbrain, DLPFC and NAc regions highlights the roles of these genes in altering their respective traits (motivation, reward memory, tolerance, and vasoconstriction) as part of the long-lasting maladaptation. In this way, an attempt was made to describe the shared genes related to substance use and how their interconnectedness may indicate shared expression, interaction, and pathways influencing maladaptation in these four brain regions, consequently leading to chronic substance exposure (Supplementary Figure 11).

### 4.3 Immediate early response genes, and EDN1, NPPA neuropeptides are differentially expressed in midbrain

The transcriptome profile of the midbrain contained genes that were mostly found to be immediate early response genes from substance use. Genes FOSB, FOSL1, FOSL2, and MYC appeared to be upregulated in the midbrain region and seemed to agree with the previous studies that opioid administration induces acetylation of the upstream chromatin of c-Fos and Fosb, aiding in their expression (Kumar et al., 2005; Levine et al., 2005; Renthal et al., 2008). The regulatory network of midbrain DEGs revealed the enrichment of transcription regulators and proteins belonging to diverse functions. This protein network was functionally enriched and embedded with neuropeptides, neurotransmitters, and their targets (Figure 6). The cascade of reactions was associated with upregulated EDN1 and NPPA neuropeptides (Figure 6). EDN1 is endothelin precursor 1 and in our study was found expressing across three regions (midbrain, DLPFC and NAc) of the brain, while NPPA is Natriuretic Peptide A was found expressing exclusive to the midbrain. EDN1 was found to be under the regulation of TEAD3, TEAD4, and VDR TFs.

### 4.4 Inflammatory signaling in midbrains

Besides these two neuropeptides, targets of DA production were also found to be upregulated along with CEBPD (Bannon et al., 2014), CXCL2 (Bannon et al., 2014), KCNE4 (Madsen et al., 2012), NNMT (Madsen et al., 2012), and TLR4 (Lin and Wang, 2024); these are among those that have been known to play a role in synaptic transmission, long-term synaptic plasticity, and reward processing during substance use. In comparison to our findings, Erickson et al. (2019) also identified upregulation of Cebpd in astrocytes, microglia, and in cell aggregates from alcohol-dependent mice. CEBPD, CXCL2, and several other chemokine ligands were found upregulated in the midbrains suggesting an activated neuroinflammatory state (Ramesh et al., 2013). Blednov et al. (2005) stimulated CXCR2 receptors by allowing them to bind rat CXCL2 ligands enabling the modulation of fast synaptic transmission and long-term synaptic plasticity via the activation of the extracellular signal-regulated kinase pathway (Figures 5, 6).

### 4.5 Role of midbrain exclusive genes in modulating SUDs phenotype

A subset of 186 DEGs were found expressing exclusively in the midbrain (Supplementary Table 2). These midbrain exclusive DEGs were found to be surrounded by DA, neuropeptides SST (somatostatin), POMC (proopiomelanocortin), NTS (Neurotensin), TAC1 (Tachykinin Precursor 1), CCK (Cholecsystokinin), VIP (Vasoactive Intestinal Peptide), and receptors belonging to adrenergic pathways (ADRB1, ADRA2C) (Bucheler et al., 2002). Somatostatin is known to contribute to emotionality and stress behaviors (Lin and Sibille, 2015; Robinson and Thiele, 2020). Upregulation of SST has been shown to participate in emotional and reward processing and cognition (Bell et al., 1995; Moller et al., 2003; Thermos et al., 2006). CEBPB, transcription regulator was differentially upregulated and showed associations with neuropeptides such as SST and others. Evidence from cell assays show the presence of Cebpb protein leading to increased transcription of the SST gene (Vallejo et al., 1995). A study by Kovacs et al. (2006) showed DA to increase substance P expression in medium spiny neurons in primary cultures implicating CEBPB in a positive feedback loop. Alterations in this feedback loop have been associated with drug addiction. CEBPB was found regulating 28 DEGs, of which several have been implicated for chronic substance use and addiction (Chen et al., 2016). While some of these genes are not well-studied but show the potential to serve as excellent candidates warranting a thorough validation.

### 4.6 Interplay between chemokines and neuropeptide-neurotransmitters in midbrain

Furthermore, this dataset included a subset of elevated expressions of chemokine ligand and receptor genes (CCL19, CXCL1, CXCL10, CXCL11, CXCL8, CXCR1). This chemokine cluster signifies neuroinflammatory signatures from neurons and glia and plays a substantial role in the modulation of midbrain DA systems (Reyes-Gibby et al., 2015). This interplay between chemokines and the neuropeptide-neurotransmitter axis signifies the role of upstream neuroinflammation following repeated substance use. Two more genes (NPY2R and OXTR) belonging to the neuropeptide-neurotransmitter axis were found differentially expressed in our study. NPY2R, Neuropeptide Y Receptor Y2, was found significantly downregulated. NPY binds to NPY2R, modulating neurobiological responses to polysubstance during GABAergic and glutamatergic transmission, and the expression levels of NPY2R and NPY have been implicated to be dysregulated during polysubstance use (Robinson and Thiele, 2017). Similarly, another neuropeptide receptor, OXTR, was found upregulated in the midbrain. Though OXTR is known to express diversely across CNS, it was found enriched in midbrains, probably hinting at an enhancement of excitability of neural cells (Stoop, 2012). Further, OXTRs are known to regulate social and emotional behaviors and reward processes (Freeman and Young, 2016). Investigation into genes exclusively expressed in the midbrain identified dysregulated genes that participated in the initiation of neuroinflammation, modulation of the DA system, mood regulation, and reward process (Figure 6).

Emerging evidence indicates that drugs of abuse perturb the balance of excitatory and inhibitory neurotransmitters and recruit stress-related neuropeptides, pushing reward circuits into an allostatic state (Volkow et al., 2019). For example, chronic exposure to ethanol and nicotine increases prepro-orexin mRNA and orexin receptor expression in the lateral hypothalamus, and orexin-producing neurons strongly activate mesolimbic dopamine and stress pathways; antagonism of orexin receptors reduces ethanol or nicotine self-administration and cue-induced reinstatement (Sharf et al., 2010). In the NAc, glutamatergic inputs drive burst firing of VTA dopamine neurons, whereas local GABAergic interneurons ordinarily restrain this activity. Opioids inhibit GABA release via mu-opioid receptors, disinhibiting dopaminergic firing; with repeated drug use, excitatory drive becomes sensitized and GABAergic tone weakens, promoting compulsive cue-driven seeking and withdrawal hyperexcitability (Volkow et al., 2019). Peptide modulators also interface with these circuits: in the NAc shell, the neuropeptide neuromedin U (NMU) dampens cocaine-induced GABA efflux, and knockdown of its receptor increases cocaine seeking (Ly and Root, 2022). Together, these findings support our interpretation that dysregulated interactions between neurotransmitters and neuropeptides contribute to the shared molecular signatures observed across midbrain, DLPFC, NAc and amygdala, and underscore the importance of considering multi-system neurochemical imbalances in SUDs.

### 4.7 ADM, EDN1 and NPAS4 modulate DLPFC during substance use

ADM is a neuropeptide that can activate signaling pathways that are upstream of immediate early gene induction, including FOSL1 and TEAD4 (Famitafreshi and Karimian, 2021) (Supplementary Figure 8). Another familiar gene, NPAS4 that was upregulated in the midbrain, was found downregulated by several folds in the DLPFC region. NPAS4 is known to be enriched throughout the brain (Ooe et al., 2004; Leong et al., 2013; Choy et al., 2015) and plays an important role in the expression of immediate early response genes such as c-Fos and BDNF participating in synaptic plasticity (Lin et al., 2008; Bloodgood et al., 2013; Spiegel et al., 2014). NPAS4 has been identified to promote normal social interaction and contextual memory, at least in the NAc region. Based on its discordant expression across brain regions, the role of NPAS4 seems to be more distributed than was previously expected. NPAS4 is known to influence the expression of BDNF and modulate GABAergic synapse development, which has been linked to SUDs (Lin et al., 2008; Luscher et al., 2011). This study identifies and acknowledges the presence of NPAS4 for the first time in three regions of the brain (midbrain, DLPFC and NAc), which appears to play an even more significant role in chronic substance use than previously expected.

### 4.8 Upregulation of GABRE and down-regulation of EGR2 in DLPFC

The subset of genes exclusively expressed in DLPFC appeared to be diverse in their function (Supplementary Figure 8). One such is, GABRE which is Gamma-Aminobutyric Acid Type A Receptor Subunit Epsilon, belonging to the GABAergic pathway and was found upregulated in the DLPFC region. GABRE is a ligand-gated ionic channel mediating quickest inhibitory synaptic transmission (Chen, 2025). Upregulation of this gene exclusive to the DLPFC may reflect dysregulated GABAergic neurotransmission. Studies have identified variants in genes belonging to GABAergic signaling, including, GABRE to mediate polysubstance dependency (Cui et al., 2012). Another gene found modulated due to substance use was EGR2. EGR2 is epithelial growth response 2, found downregulated in the DLPFC region. In contrast, studies have found induction of Egr2 during exposure to polysubstance (Saint-Preux et al., 2013; Gao et al., 2017). Besides this, Egr2 is necessary for the full development of CPP. Egr2 is also part of a ‘core transcriptome’ consisting of Btg2, Egr2, Egr4, Fos, FosB, JunB, and Nr4a1 that are also reported to be explicitly expressed in the NAc region (Savell et al., 2020). The collective action of this core transcriptome is thought to increase the excitability of spiny projection neuron (SPN), leading to enhanced development of cocaine sensitization (Savell et al., 2020).

### 4.9 Upregulation of opioids, cannabinoids, and DA receptors (RGS proteins) exclusive to DLPFC

Further, two RGS (Regulator of G protein-signaling) family proteins, RGS1 and RGS16, belonging to the R4 family of RGS proteins, were found upregulated in the DLPFC region. These proteins function as receptors for opioids, cannabinoids, and DA and were also found upregulated after amphetamine stimulation in the rat (Traynor, 2010). RGS13, (RGS16 in case of amygdala) has been reported in DLPFC, NAc, and midbrain regions of rat studies with chronic nicotine exposure (Burchett et al., 1998; Burchett et al., 1999; Konu et al., 2001). In contrast, our study identified differential upregulation of RGS proteins for the first time, paving the way for further validations. RGS proteins play a crucial role in psychostimulant and opiate dependence via activation of the PKC pathway and show diverse distribution patterns and appear to modulate distinct signal transduction events involving opiate and psychostimulant drug reward (Bowers, 2010) (Supplementary Figure 8).

### 4.10 DEGs of NAc enriched in synaptic transmission

The transcriptome profile of NAc was found comparable with that of the midbrain transcriptome than with the DLPFC’s profile based on the number of overlapping genes in DEGs. CXCL2, differentially upregulated in the midbrain region, continued to be differentially upregulated even in the NAc region. A cluster of FcγR genes, FCGR 1A, 1B, 2A, 3A (Fc fragment of IgA receptor and Fc fragment of IgG receptors Ia, Ib, IIA, IIIa) along with FCRLA (Fc receptor like A) were found upregulated in the NAc region. Increased expression of FcγR in the NAc has been shown to have the potential to induce inflammatory signals and modulate long-term synaptic transmission (Fuller et al., 2014). While RGS proteins mediated G protein-signaling were found exclusively in DLPFC regions (except for GPR4 in amygdala), GPR proteins (G protein-coupled receptor), encoded by GPR4, GPR15, GPR84, and GPRC5A genes were found up-regulated in NAc (Supplementary Figure 9). G protein-coupled receptors function as neurotransmitter receptors and are known to perform slower neuromodulatory actions (Rosenbaum et al., 2009). Substance use intervenes with the receptor functioning altering neuromodulation, thus paving the way for induction signals towards reward and dependency (Johnson and Lovinger, 2016). Though GPRs are familiar in their role in addiction manifestation, it needs to be seen whether the related genes GPR4, GPR15, GPR84, and GPRC5A, currently identified in the study also follow the same pathway towards development of addiction.

### 4.11 Role of DEGs exclusive to NAc in SUDs

NAc region expressed 160 transcripts that were exclusive and not found expressed in any of the other three regions (Supplementary Table 2, Supplementary Figure 9). Complement receptor genes, CR1 and CR2, were found differentially upregulated along with Fc fragments for IgA and IgG receptors. A growing body of evidence has convincingly provided evidence for the role of the complement system in the regulation of neural plasticity (Gong et al., 2013; Perez-Alcazar et al., 2014; Stokowska et al., 2017). Complement proteins, C3 and C1q, have been shown to localize to synapses that are marked for elimination. Animal studies involving knockout of either C3 or C3 receptor show a significant disruption in synaptic pruning (Schafer et al., 2012; Lacagnina et al., 2017). These results provide compelling evidence that glial cells mediate neural synapse alteration. Further, discordant expressions of complement protein-coding genes such as downregulation of C1QL3 in midbrain and upregulation of C1R in amygdala were also detected. Solute carrier genes were also found exclusive to NAc and were mostly downregulated (SLC17A6, SLC18A3, SLC2A5, SLC38A8, SLC6A7, SLC9A4, and SLCO4C1) except for one (SLC2A5) (Supplementary Figure 9). These solute carriers are transmembrane transporters that permit the exchange of ions, drugs, and metabolites (Hu et al., 2020). Downregulation of solute transporters has been shown to create an imbalance in ionic concentration, exchange, and distribution subsequently altering the efficacy of neuropeptide and neurotransmitter transportation (Vaughan, 2004).

### 4.12 Downregulation of NGFR, NPAS1, NXPH4, and NCALD in NAc

This study identified significant downregulation of genes NGFR, NPAS1, NXPH4, and NCALD in the NAc region. NGFR, associated with nerve growth factor signaling, has been linked to epigenetic regulation during alcohol withdrawal and affected by substance exposure (Heberlein et al., 2013). NPAS1 is involved in psychosis-related inhibitory interneuron regulation, with knockouts displaying behavioral abnormalities (Erbel-Sieler et al., 2004). NXPH4 regulates synapse functions, and its downregulation suggests potential implications in substance use disorders (Meng et al., 2019). NCALD’s negative dysregulation points to its possible role in addiction, given its involvement in signal transduction (Kim et al., 2021). Upregulated chemokine signaling genes CCL8 and CXCR2, and downregulated neurotransmitter receptor genes ADRA2A, CHRNA6, HCRTR2, and PRLHR in the NAc, further delineate the complex molecular landscape of substance addiction, encompassing neuroinflammation, pain, and neurotransmitter regulation (Comasco et al., 2015).

### 4.13 NAc shows dysregulated cholinergic and nociceptive signaling

The current study highlights the critical role of cholinergic receptors (nAChRs), specifically CHRNA6, and their influence on DA neurotransmission in addiction pathways, with CHRNA6 found downregulated in the NAc region (Papke et al., 2020). The findings emphasize nAChRs’ modulatory function on neurotransmitters, suggesting potential for further investigation in chronic substance use. Additionally, the orexin/hypocretin system, via HCRTR2 receptors, plays a pivotal role in behaviors like feeding, sleep, and arousal, with dysregulation observed in the NAc (Sharf et al., 2010). This supports the involvement of orexins in reward and addiction pathways due to their widespread brain expression and specific downregulation in key areas associated with addiction. Moreover, we found downregulation of the PRLHR receptor, which is expressed in pain-processing circuits and is implicated in nociceptive signaling and pain modulation, likely by influencing central processing of nociceptive inputs rather than serving as a primary peripheral nociceptor. Mice studies with the knockout of PRLHR showed higher nociceptive thresholds and stronger stress-induced analgesia than wild-type mice (Laurent et al., 2005). Acute nociceptive pain is regulated by nociceptors involved in sensory, emotional, and reward functions (Elman and Borsook, 2016). This important role mediated by nociceptors from initiation to transition from regular use to the development of addiction may hold the basis for establishing dependency.

### 4.14 Transcription regulators of amygdala

Amygdala region shared some similarities with midbrain with regards to the upregulation of immediate early response genes such as MYC, and FOSL1 (Supplementary Figure 10). These two IEGs are central to the amygdala’s transcriptomic dynamics in relation to SUDs. Besides these two, MAFF, YBX3, CEBPD, TEAD4, ZFP36, and NFKB2 are another set of transcription regulators found differentially expressed in amygdala. MAFF, upregulated in the amygdala region is known to bind and interact with the upstream promoter regions of the oxytocin receptor gene and behaves as a transcriptional enhancer leading to its up-regulation. Oxytocin has been shown to establish extensive cross-talks with the neural circuits involved in motivational stimuli by enabling dopaminergic neurotransmission (Brinton et al., 1984; Ross et al., 2009). Oxytocin secreted from hypothalamus, makes its way in to amygdala, hippocampus, NAc, and VTA possibly through diffusion thus stimulating dopamine neurons facilitating reward and motivated behavior (Stoop, 2012). Similarly, YBX3, another upregulated transcriptional regulator is known to bind and interact with G-CSF promoter (CSF3 in this case). Calipari et al. (2018) identified G-CSF as a potent mediator of cocaine-induced adaptations in the NAc and DLPFC regions. G-CSF promoters have a unique role in the activation of microglia, including the potential to induce neuronal network dysfunction (Basso et al., 2017; Stanley et al., 2023). Since similar expression signatures are also found in the amygdala region, it is safe to assume that the dysfunction possibly extends up to the amygdala region that was previously limited to NAc and DLPFC regions. Furthermore, this activation status extends beyond to include the targets, BCL2A1 and NFKB, both found upregulated in the amygdala regions. BCL2A1 gene has been considered a direct transcription target of NF-kappa B in response to inflammatory mediators and is known to be up-regulated by G-CSF (Zong et al., 1999; Vogler, 2012) (Supplementary Figure 10).

### 4.15 Role of DEGs exclusive to amygdala in SUDs

Our findings elucidate pivotal roles for Neuropeptides B and W receptor 2 (NPBWR2) and Nuclear Factor Kappa B Subunit 2 (NFkB2) within the opioid signaling pathway, both showing significant upregulation in the amygdala (Supplementary Figure 10). Neuropeptides W and B, acting as ligands for NPBWR2, initiate downstream signaling processes, whereas ATF3 directly modulates NFkB2 activity. Importantly, NFkB2 is implicated in the regulation of corticotropin-releasing hormone and lymphotoxin alpha (LTA), thus providing a mechanistic insight into the neuroimmune interactions underlying SUDs (Wang et al., 2012, 2013; Yang et al., 2022). Our analysis further underscores NFkB2’s broader regulatory scope, encompassing genes associated with addiction, learning, memory, stress response, and drug reward (Nennig and Schank, 2017). Glucocorticoids has been shown to interact with regulatory elements of tyrosine hydroxylase (TH) causing upregulation of TH and subsequently increasing DA secretion (Navarro-Zaragoza et al., 2020). Interestingly, we report the upregulation of RIPPLY3, a transcriptional repressor that influences the expression of several genes (IL1RN, CPA4, CD207, ORM1, CASP14, IGHG2) involved in neuroimmune responses, with corresponding downregulation of LCN2. Notably, the absence of Il1rn has been linked to reduced ethanol consumption and preference in mice (Blednov et al., 2012), suggesting a potential target for therapeutic intervention. However, the specific interactions between RIPPLY3 and other genes within the context of SUDs and their impact on the amygdala’s function remain to be fully elucidated.

### 4.16 THBS1 and TLR-2 maintain substance-induced synaptic adaptations for craving and reward memory in tripartite regions

Another gene that has shown the potential of a good target for further exploration is THBS1 (Thrombospondin 1). This gene found upregulated in both midbrain, NAc, and amygdala regions is known to promote synaptogenesis that is presynaptically active but postsynaptically silent, indicating midbrain, NAc, and amygdala regions are possibly receiving induction signals from substance use (Christopherson et al., 2005; Risher and Eroglu, 2012). Astrocytes localized in the midbrain, NAc, and amygdala may mediate synaptogenesis towards forming excitatory synapse formation (Eroglu et al., 2009). By consistently finding THBS1 differentially regulated across brain regions in the current study and given its role in forming synapses, we emphasize its relevance in astrocytic signaling mediated, substance-induced synaptic and circuit adaptations towards the establishment of craving and reward memory. Besides these astrocyte-mediated synapses, glial cells utilize toll-like receptor signaling to mediate synaptic plasticity. TLR-2 gene was found upregulated in midbrain, NAc, and amygdala regions and revealed the participation of innate immune system in chronic substance users. Increased TLR-2 expression has also been documented in the human alcoholic orbifrontal cortex (Crews et al., 2013; Vetreno et al., 2013). Similarly, studies on mice have also shown concordant results of increased Tlr-2 expression due to ethanol induction. Therefore, a steady state of TLR signaling and innate immune activation has been shown to contribute towards brain alterations laying the groundwork for the development of addiction (Liu et al., 2011; Pascual et al., 2015; Montesinos et al., 2016).

Our DGE analysis incorporated covariate regression for age, sex, brain pH and RNA integrity number to reduce technical confounding. Subjects with documented psychiatric comorbidities were excluded from the cohorts, which minimizes confounding from other mental-health conditions but may limit the generalizability of the findings to populations with co-occurring disorders. The TFs and regulatory networks predicted by IPA are based on curated knowledge and statistical enrichment; functional experiments will be needed to confirm that these regulators modulate the observed gene expression changes. Finally, our observation of a neuropeptide-neurotransmitter imbalance arises from co-expression patterns and literature-derived interactions rather than direct measurements; these associations should be considered hypothesis-generating rather than causal evidence for SUDs. To summarize, the current study has made the following new observations; about 25% of the DEGs were shared among the tetrapartite regions, and presence of both unique and shared expression signatures for each tetrapartite region were found, thus implying that induction and exclusion signals were driving distinctive pathway signaling, and sustained addiction behavior. Shared genes such as CSF3, GADD45B, SOCS3, and EDN1 are upregulated in all regions possibly involved in driving motivation, reward memory, tolerance, and vasoconstriction due to chronic substance use. NPAS4 on the other hand was comparatively both upregulated and downregulated indicating the altered role of reward-related behavior. This study, while recognizing known genes in the field of substance biology and addiction, also identified probable new gene candidates and biomarkers that confer susceptibility towards addiction risk and maladaptations. These findings and observations would pave the way to help understand the intricacies of alterations in the human brain function due to chronic substance use, aiding in advancing our understanding of each of the described stages of substance use and provide potential targets for therapeutic intervention.

## Data Availability

The original contributions presented in the study are included in the article/Supplementary material, further inquiries can be directed to the corresponding author.

## Supplementary material

SUPPLEMENTARY FIGURE 1 Details of the cohorts and brain regions included in the current study.

SUPPLEMENTARY FIGURE 2 Total read counts (millions) for each cohort. **(a)** Is midbrain cohort, **(b)** is DLPFC cohort, **(c)** is NAc cohort, and **(d)** is amygdala cohort.

SUPPLEMENTARY FIGURE 3 Shows basic QC of distribution of transformed data, and density plot of transformed data in **(a)** midbrain cohort, **(b)** DLPFC cohort, **(c)** NAc cohort and d. amygdala cohort.

SUPPLEMENTARY FIGURE 4 Illustrates the predicted mechanistic interactions between the top regulators identified in the upstream regulator analysis of IPA and the DEGs that are commonly affected across all four brain regions.

SUPPLEMENTARY FIGURE 5 Shows gene expression signatures of DLPFC cohort. **(a)** Is heatmap, **(b)** is a correlation matrix, **(c)** is the gene dendrogram and respective module colors and **(d)** shows the lollipop plot based on WGCNA.

SUPPLEMENTARY FIGURE 6 Shows gene expression signatures of NAc cohort. **(a)** Is heatmap, **(b)** is a correlation matrix, **(c)** is the gene dendrogram and respective module colors and **(d)** shows the network and **(e, f)** shows the lollipop plot, both based on WGCNA.

SUPPLEMENTARY FIGURE 7 Shows gene expression signatures of amygdala cohort. **(a)** Is heatmap, **(b)** is a correlation matrix, **(c)** is the gene dendrogram and respective module colors and **(d)** shows the lollipop plot based on WGCNA.

SUPPLEMENTARY FIGURE 8 Shows the regulatory and protein-protein interaction network based on DEGs from the DLPFC region overlayed with log2 fold-change and *p*-value. Several DEGs directly interacted with neuropeptides and DA targets. EGR2, a downregulated transcription regulator, was found to control several neuropeptides and neurotransmitters, targeting CHRNA7, GRIN2A, GRIA2, PTHLH, CALCB, GHRH, IGF2, IGF1, and SOCS3. These, in turn, regulated some of the DEGs. Two other significant hubs in the network were centered upon DEGs, CEBPD, and ZFP36. CEBPD regulated neuropeptides such as CRH, AGT, LEP, and IGF1, and it regulated neurotransmitter receptor ADRB3 and other DEGs such as BCL2A1, CD14, HAMP, and CDKN1A. CEBPD was found to be under upstream DEGs, IL6, and IL1B. In contrast, the ZFP36 hub regulated only DEGs LIF, SPP1, CDKN1A, IL1B, RGS16, S100A8, THBD, and S100A9; these DEGs in turn regulated neuropeptides CRH, NPPA, CHGB, and DA target DRD2. EDN1 and ADM neuropeptides interacted with other neuropeptides and neurotransmitters, such as EDN3, NPPB, and serotonin (5-hydroxyltryptamine). These further regulated a plethora of neuropeptides and neurotransmitters (and their targets) that play a role in SUDs. Besides these, CCL2 was also found interacting with serotonin (5-hydroxyltryptamine).

SUPPLEMENTARY FIGURE 9 Shows the IPA regulatory network of differentially expressed genes from NAc region overlayed with log2 fold-change and *p*-value. TEAD4, CEBPA, and ZFP36 were found regulating the entire neighborhood of the network. TEAD4 directly regulated differentially expressing neuropeptides EDN1, EDN3, CCK, ADM, and ADIPOQ, besides regulating CEBPA. CEBPA is a transcription regulator found downregulated and appeared to regulate a host of differentially expressed downstream transcription regulators NFIL3, GRHL3, and OTX2 besides regulating neuropeptides AGT, TAC1, LEP, ADIPOQ, GAL, APLN, IGF1, and RETN. Another regulatory hub was led by ZFP36, and it regulated GABAergic and glutamatergic receptor clusters via the upregulation of MEFV. Mir-637 was found to regulate a combination of DEGs and neuropeptides, among which NPAS1 was a prominent one. Mir-637 was found centrally located and upregulating genes LIF, HAMP, CISH, TRIM63, MYBPH, CD300E, CD209, APOL4, GADD45B, SECTM1, and IL4R, while downregulating genes NGFR, CEBPA, TRIM54, SUSD2, REM1, SCUBE1, GALNT9, CCDC177, RTBDN, and NXPH4.

SUPPLEMENTARY FIGURE 10 Shows the IPA regulatory network from the amygdala region overlayed with log2 fold-change and *p*-value. The network density of the amygdala region was comparable with other regions mainly with the number of DEGs around the neighborhood axis of neuropeptides and neurotransmitters. The upregulated MYC was found to further regulate the expression and function of EDN1, FOSL1, CEBPD, THBS1, and GRHL3. MYC was found under the upregulation of CD44, IL6, NFKB2, CEBPD, ZFP36, CDKN1A, and PIM1. Further, TEAD4, SNAI1, YBX3 continued the regulatory signaling that was initiated by MYC. ADM neuropeptide was found regulated by FOSL1, TEAD4 and MYC. CSF3 was found under the influence of FOSL1 while influencing the downstream neuropeptide effectors such as EDN1. MYC, and FOSL1 were found to be central to the amygdala’s transcriptomic dynamics. Besides these two, MAFF, YBX3, CEBPD, TEAD4, ZFP36, and NFKB2 were the next set of transcription regulators that were crucial towards the network. MAFF upregulated in the amygdala region is known to bind and interact with the upstream promoter regions of the oxytocin receptor gene. Similarly, YBX3, another upregulated transcriptional regulator is known to bind and interact with CSF3. Furthermore, this activation status extends beyond to include the targets BCL2A1 and NFKB, both found upregulated in amygdala regions. BCL2A1 gene has been considered a direct transcription target of NF-kappa B in response to inflammatory mediators and is known to be up-regulated by G-CSF.

SUPPLEMENTARY FIGURE 11 Shows the network of 44 genes that appeared to be expressing across all four regions. **(a)** Network shows the cross talk that exists among the commonly expressing genes. **(b)** Network showing the cross talk in a neuropeptide-neurotransmitter axis. Some of the notable genes in these networks were CSF3, CCL2, CD14, CP, EDN1, and GADD45B, among other lesser-known candidates like ANGPTL4, BCL2A1, CISH, DEPP1, EMP1, FPR1, FCGR1A, MAFF, IL6, IL1B, HAPMP, and FPR2.
