# Supplementary figures and images for: Transcriptomic signatures in tetrapartite brain region identifies shared and unique gene signatures for substance-use
Source: Front Cell Neurosci. 2026 Mar 18;20:1770214. doi: 10.3389/fncel.2026.1770214 (PMC13038532; doi:10.3389/fncel.2026.1770214)

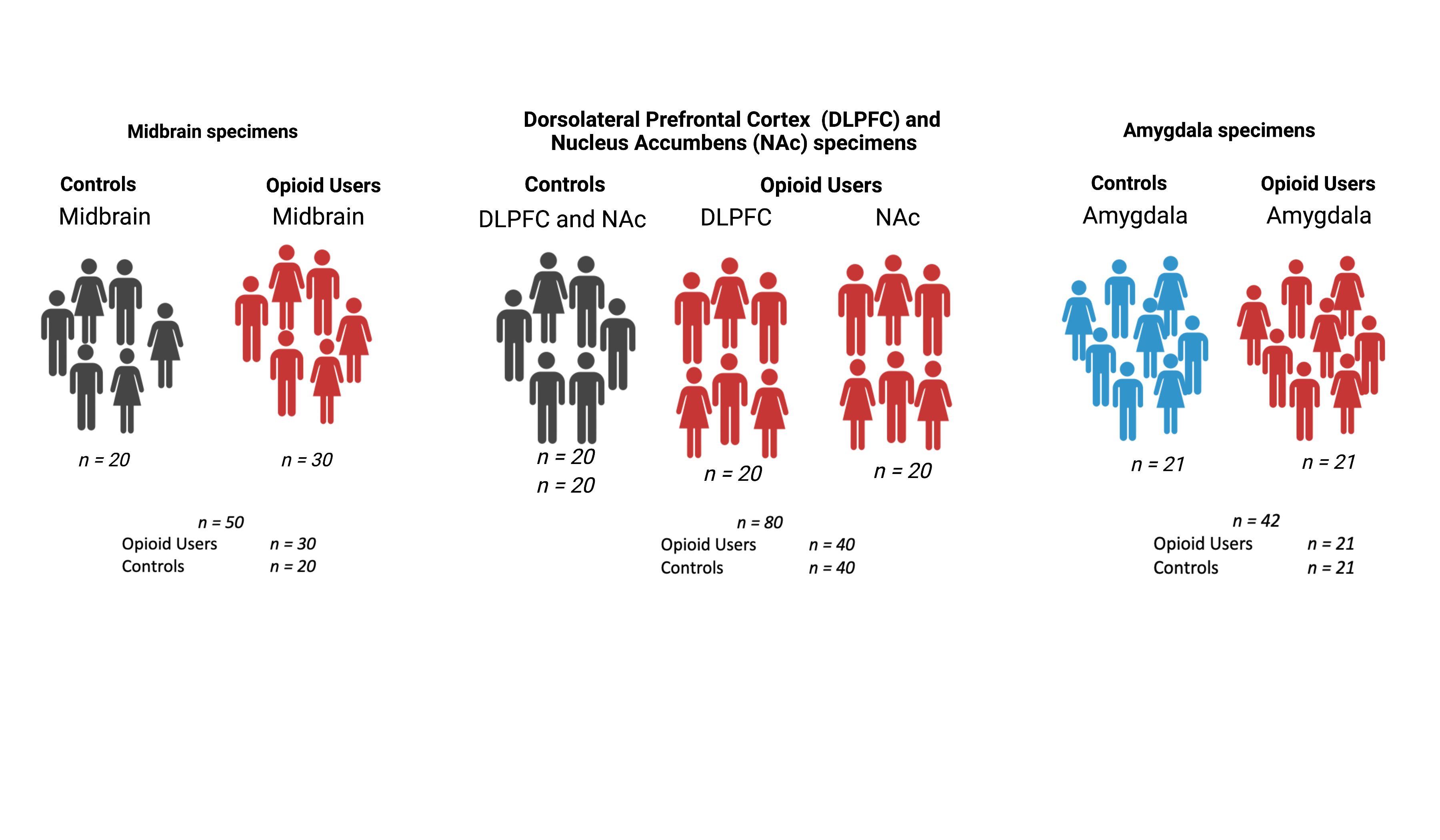

Supplement: Supplementary file 1 [file Image_1.png]

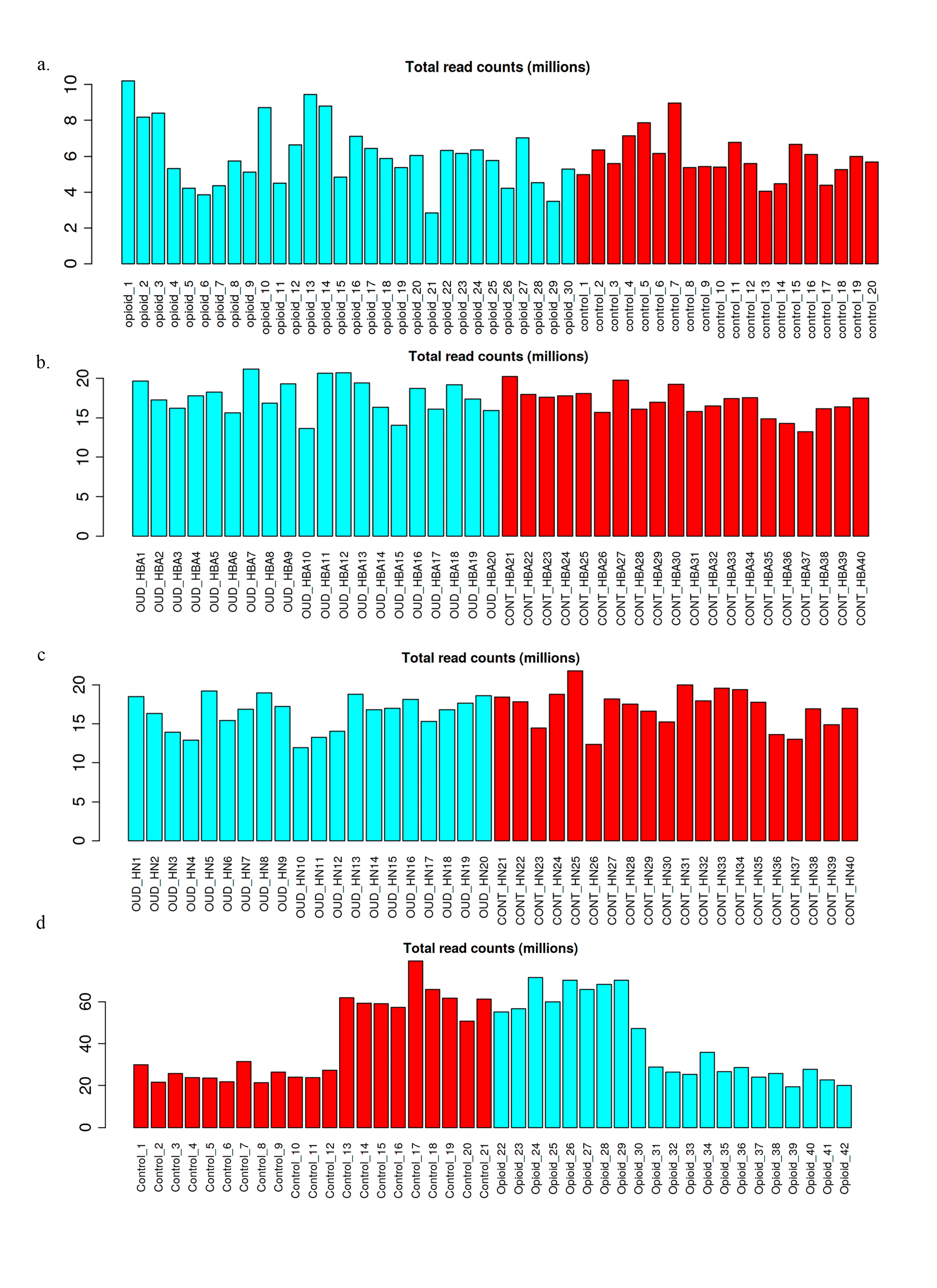

Supplement: Supplementary file 2 [file Image_2.png]

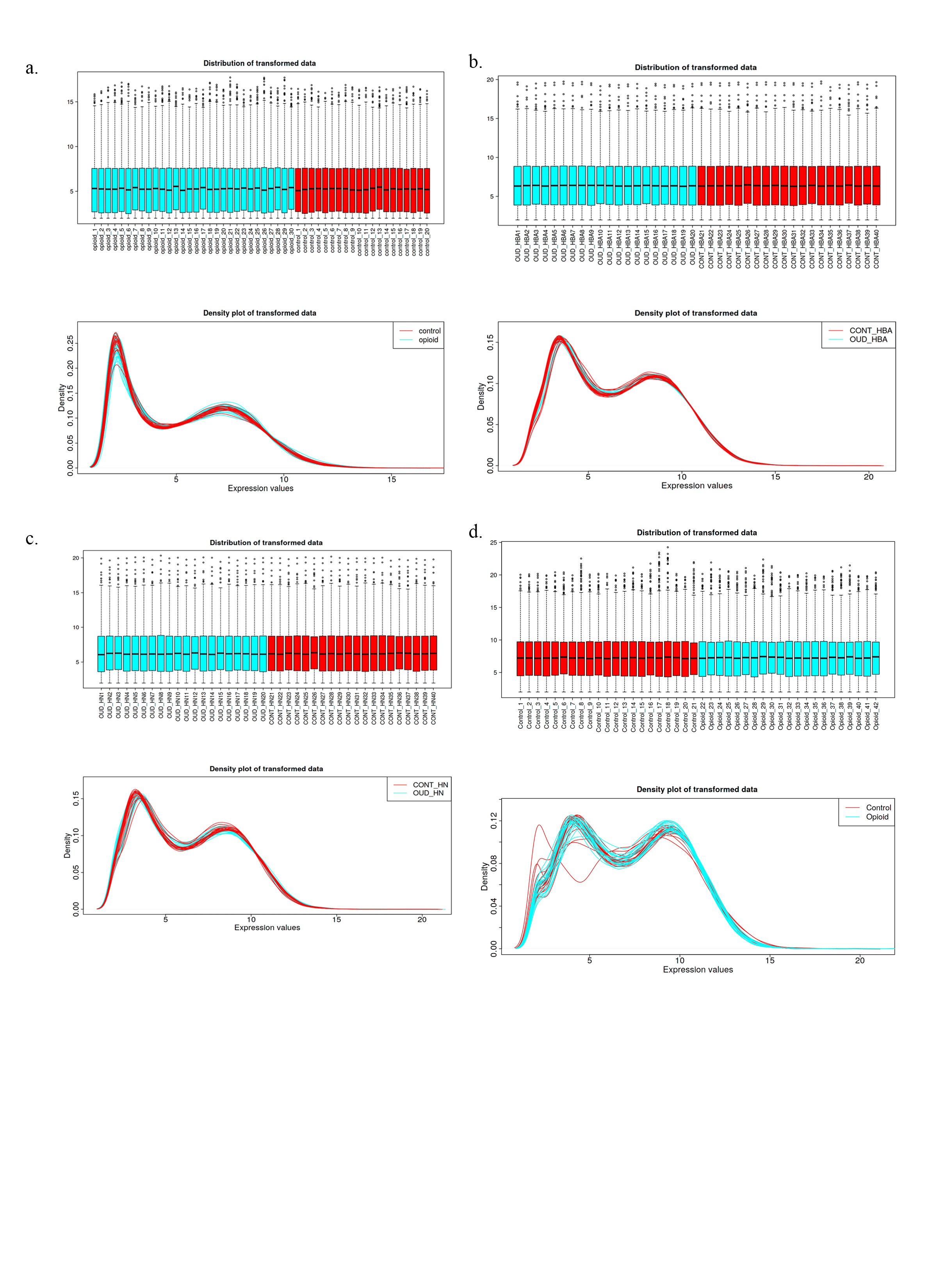

Supplement: Supplementary file 3 [file Image_3.png]

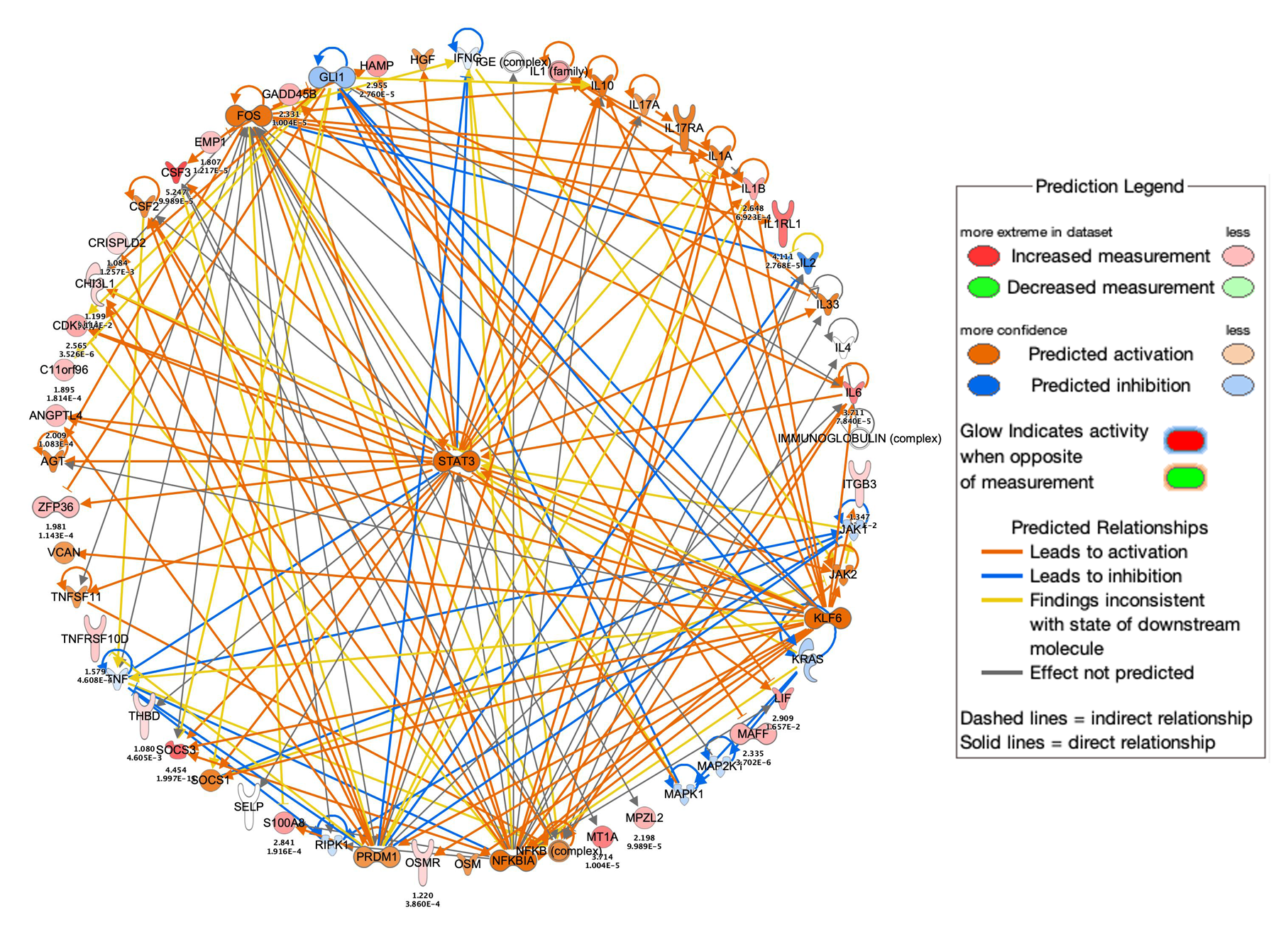

Supplement: Supplementary file 4 [file Image_4.png]

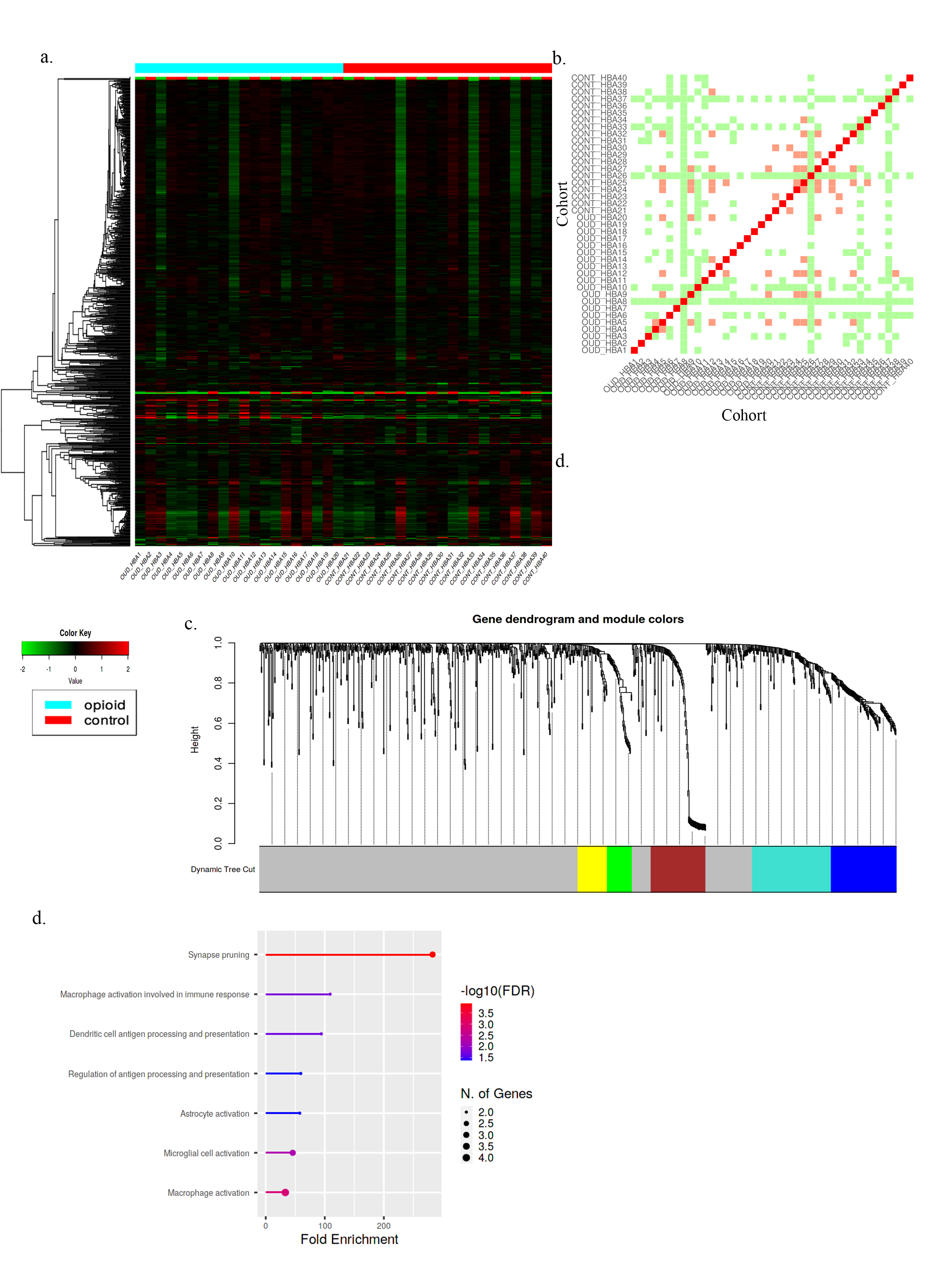

Supplement: Supplementary file 5 [file Image_5.png]

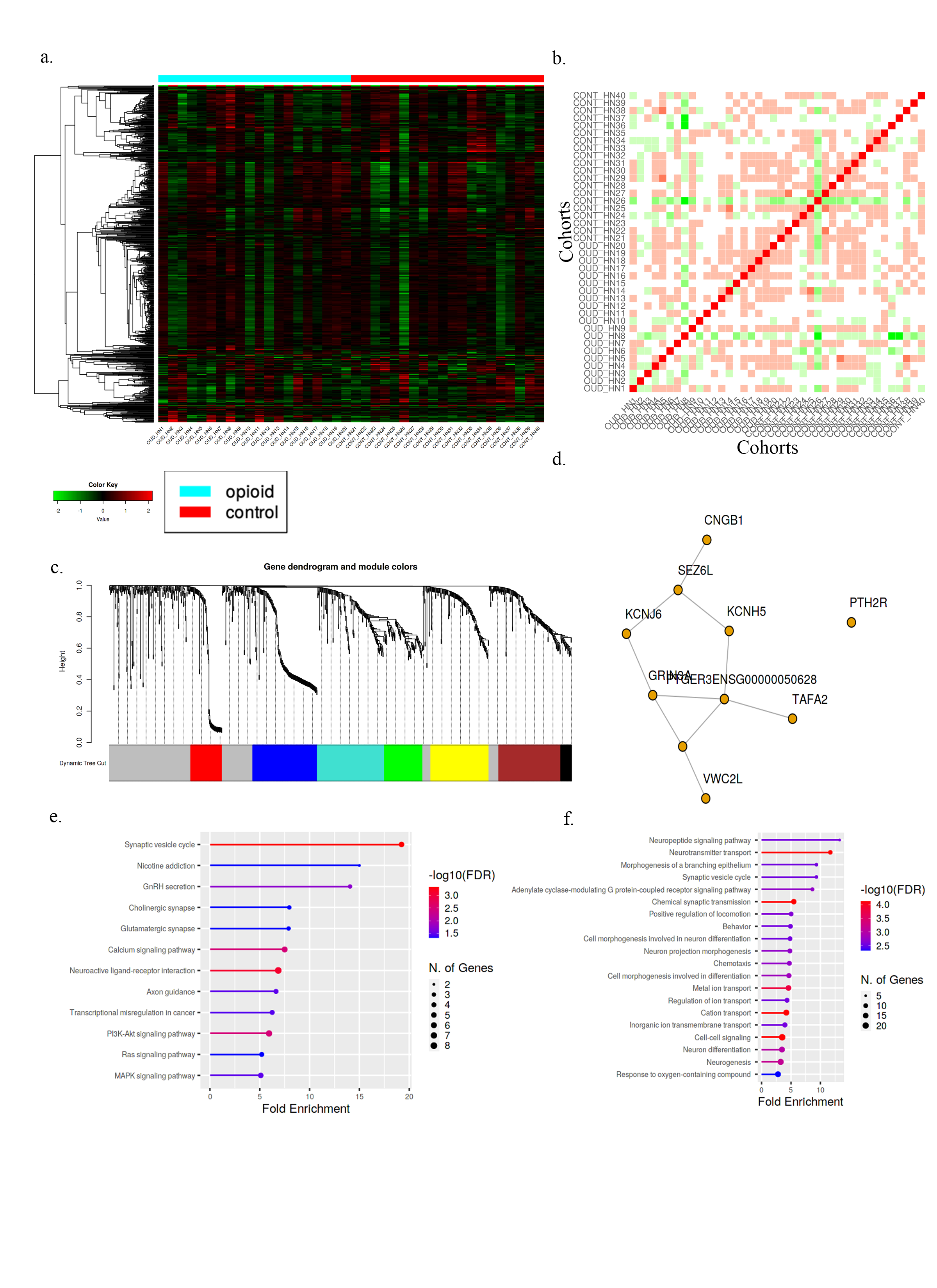

Supplement: Supplementary file 6 [file Image_6.png]

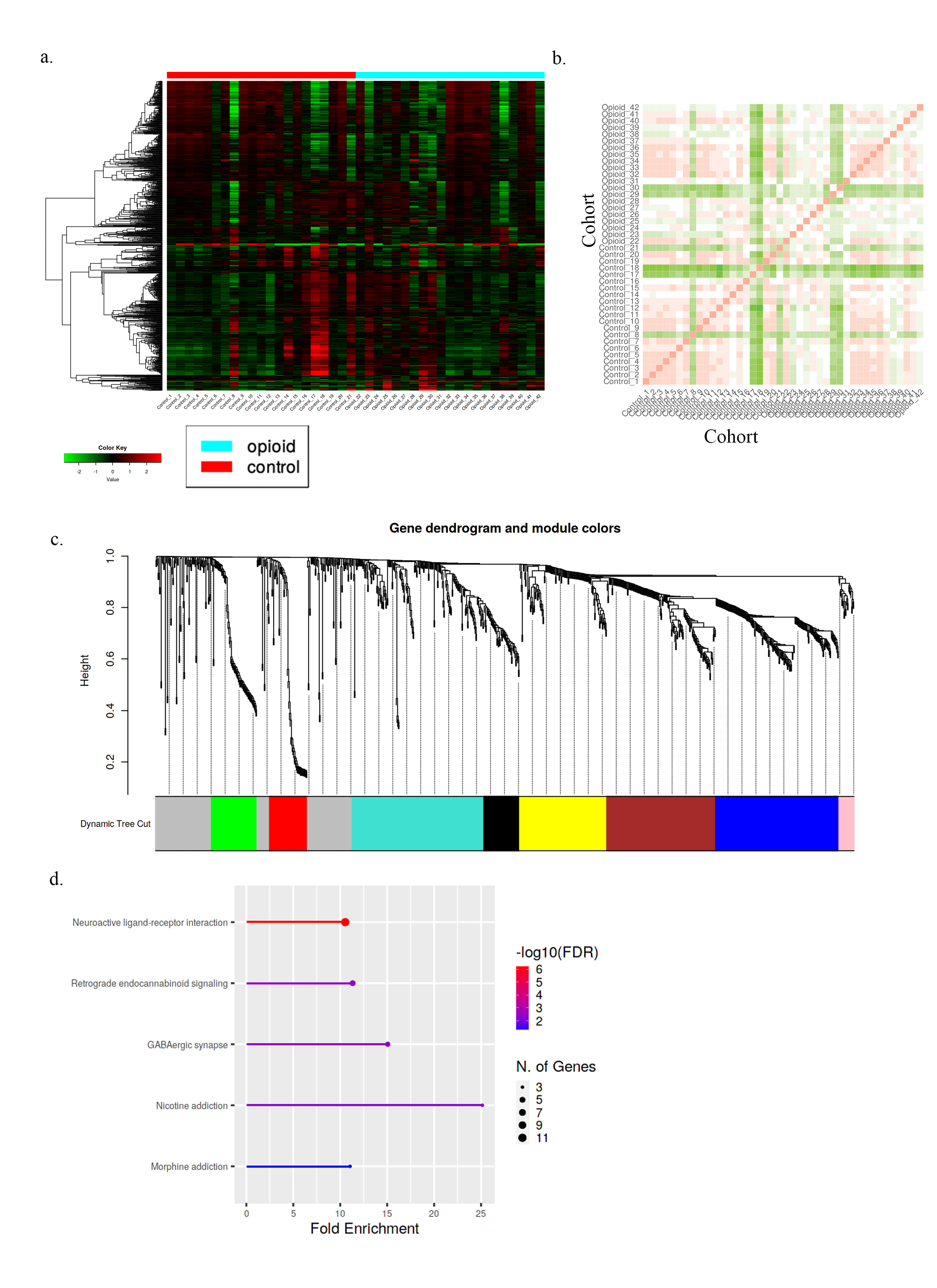

Supplement: Supplementary file 7 [file Image_7.png]

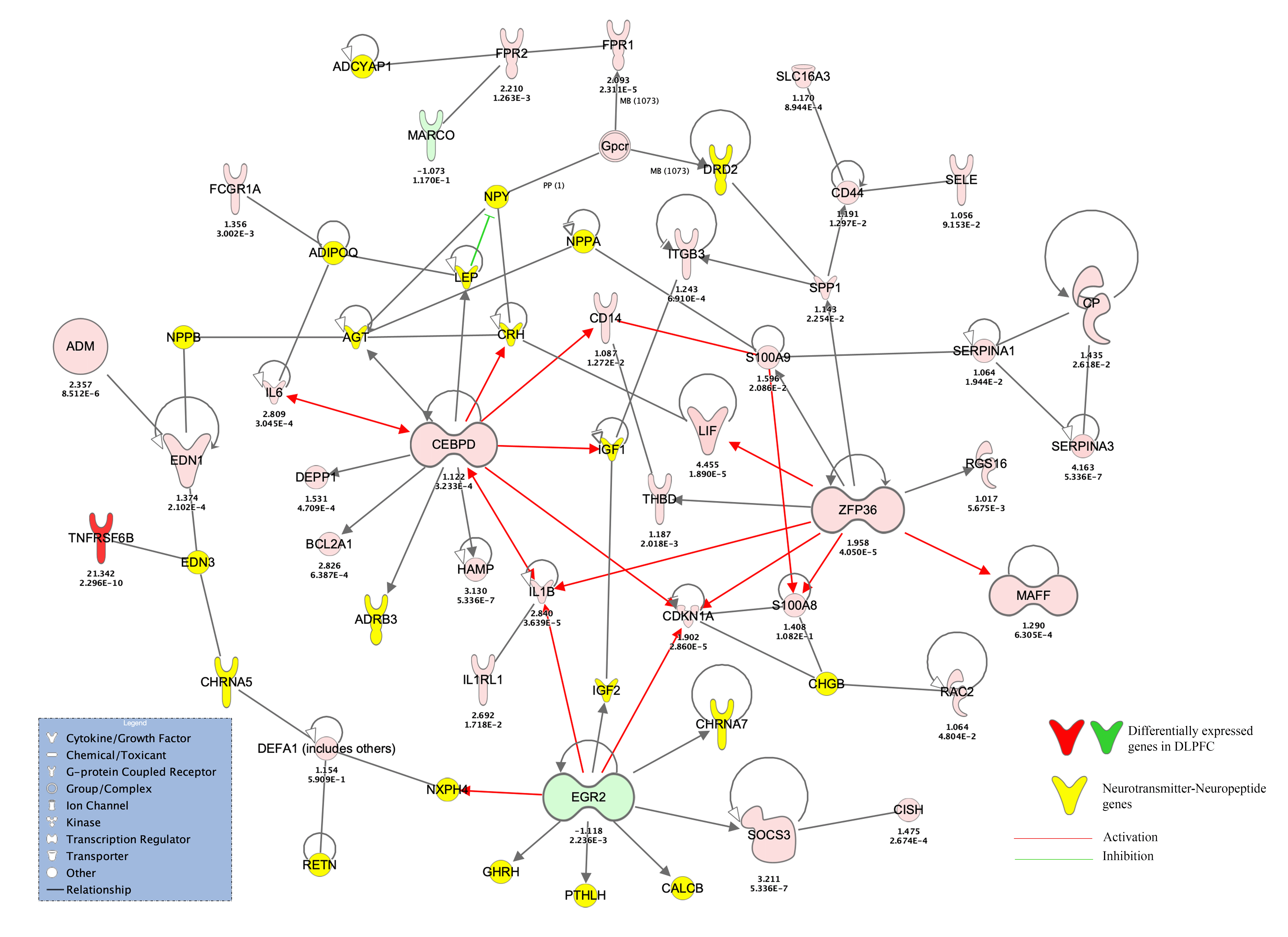

Supplement: Supplementary file 8 [file Image_8.png]

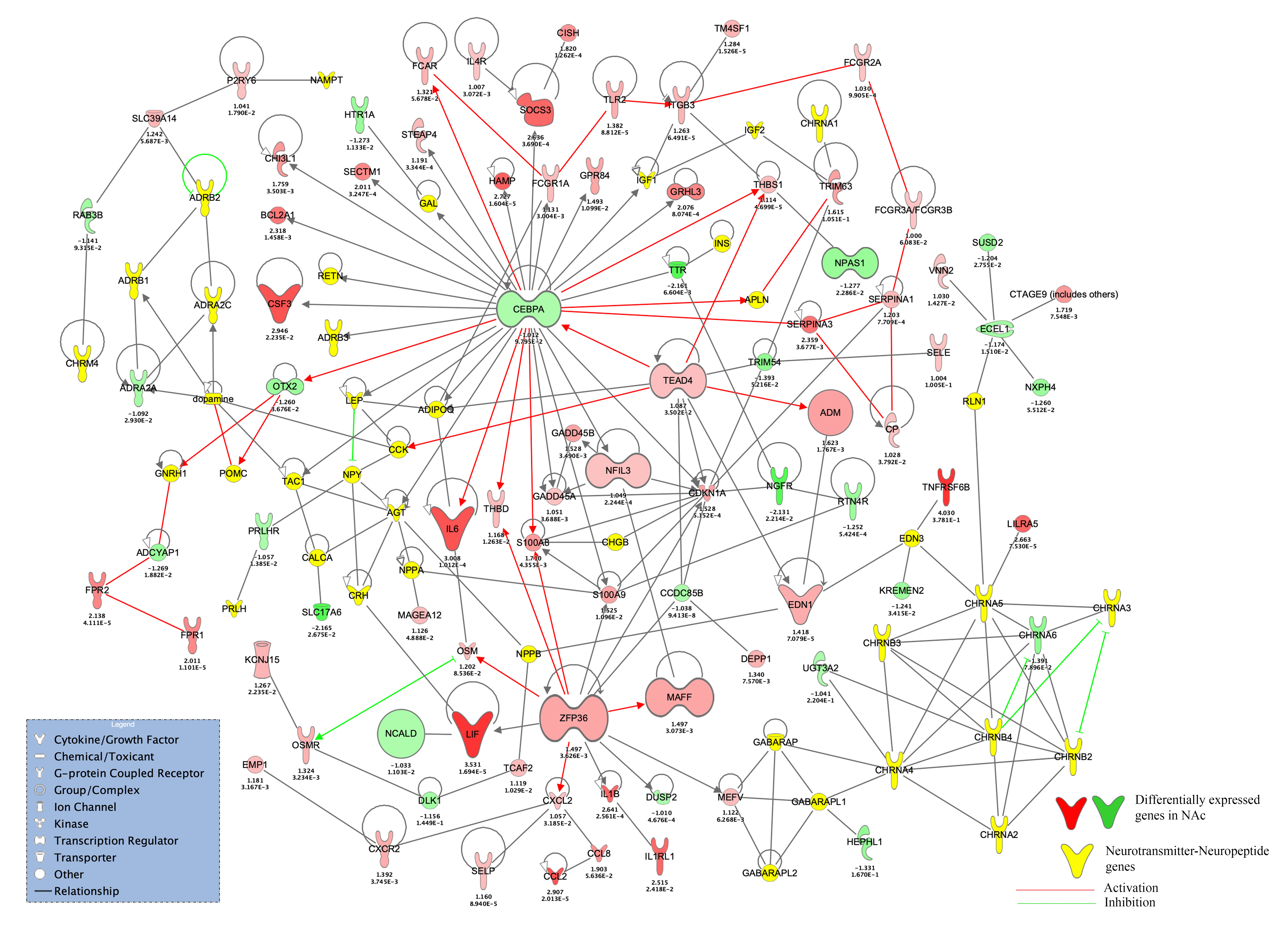

Supplement: Supplementary file 9 [file Image_9.png]

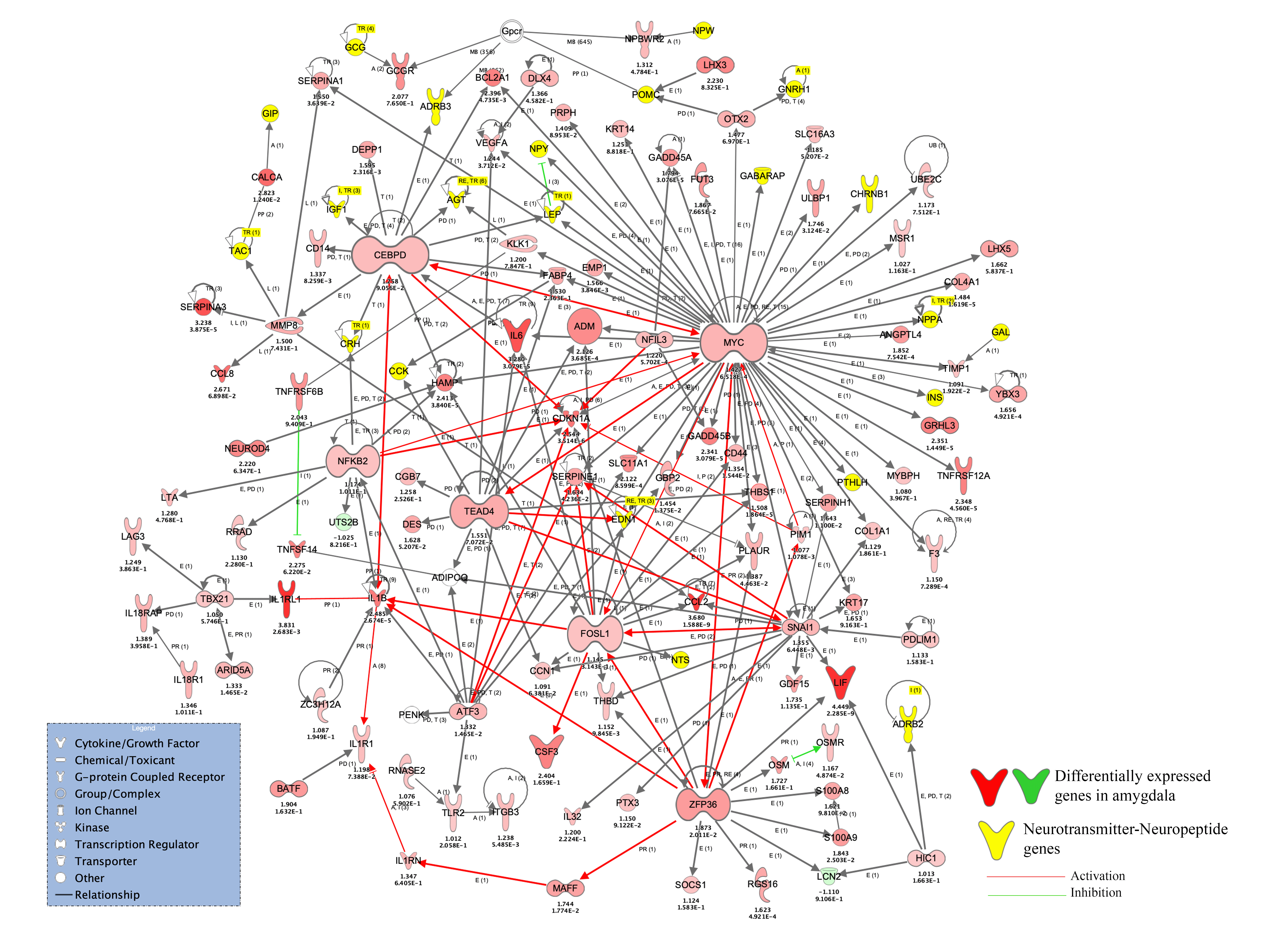

Supplement: Supplementary file 10 [file Image_10.png]

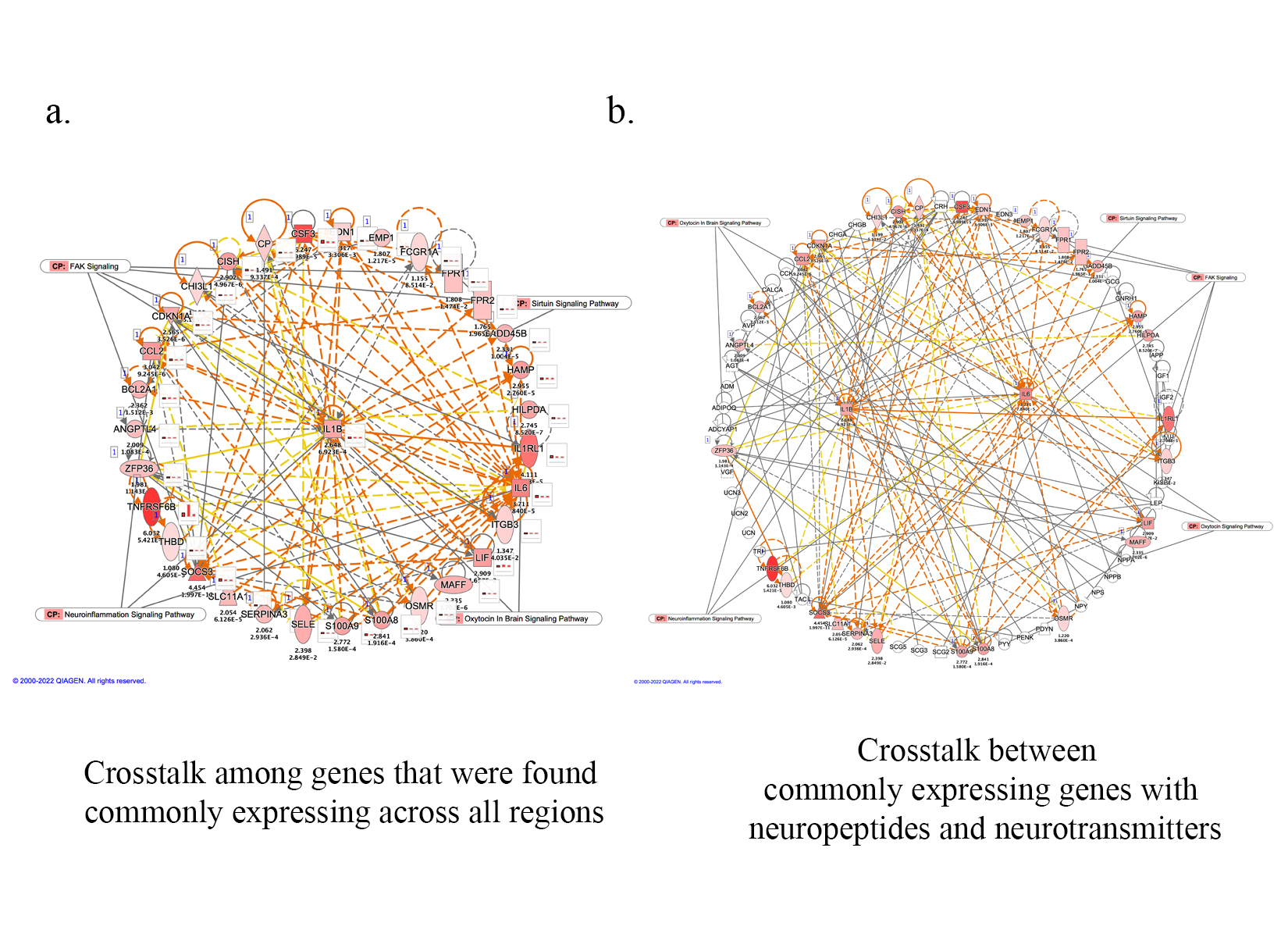

Supplement: Supplementary file 11 [file Image_11.png]
